# Effects of miR-103a-3p Targeted Regulation of TRIM66 Axis on Docetaxel Resistance and Glycolysis in Prostate Cancer Cells

**DOI:** 10.3389/fgene.2021.813793

**Published:** 2022-02-08

**Authors:** Qiang Yi, Junfeng Wei, Yangzhou Li

**Affiliations:** Department of Urology, Zhengzhou Central Hospital Affiliated to Zhengzhou University, Zhengzhou, China

**Keywords:** prostate cancer, docetaxel resistance, glycolysis, MiR-103a-3p, TRIM66

## Abstract

**Objective:** We aimed to study the expressions of miR-103a-3p and TRIM66 in prostate cancer (PCa) cells, explore the direct target genes of miR-103a-3p, and analyze the effects of miR-103a-3p targeted regulation of the TRIM66 axis on docetaxel (DTX) resistance and glycolysis of PCa cells.

**Methods:** Human normal prostate cells and PCa cells were used to detect the expressions of miR-103a-3p and TRIM66 and analyze their relationship. DTX-resistant (DR) PCa cells were established and transfected with miR-103a-3p and TRIM66 plasmids. The MTT assay, the plate cloning assay, the wound healing assay, and the Transwell assay were used to detect cell viability, colony formation, cell migration, and cell invasion, respectively. Cell glycolysis was analyzed using a cell glycolysis kit.

**Results: **The expression of miR-103a-3p was low and that of TRIM66 was high in PCa cells. MiR-103a-3p had a binding site with TRIM66, and the double luciferase report confirmed that they had a targeting relationship. Compared with the PCa group cells, the DTX-resistant group cells showed increased resistance to DTX. The resistance index was 13.33, and the doubling time of the DTX-resistant group cells was significantly longer than that of the PCa group cells. The DTX-resistant group showed more obvious low expression of miR-103a-3p and high expression of TRIM66. After the DTX-resistant group cells were transfected with miR-103a-3p and TRIM66 plasmids, the expression of miR-103a-3p increased significantly and that of TRIM66 decreased significantly. Upregulation of miR-103a-3p and interference with TRIM66 can inhibit the proliferation, metastasis, and glycolysis of DTX-resistant cells.

**Conclusion:** The expression of miR-103a-3p was downregulated and that of TRIM66 was upregulated in the malignant progression of PCa, especially during DTX resistance. Upregulation of miR-103a-3p and interference with TRIM66 can inhibit DTX resistance and glycolysis of PCa cells. Targeting TRIM66 may provide potential application value in molecular therapy for PCa.

## Introduction

Prostate cancer (PCa) is one of the fatal malignant tumors and has become the second leading cause of adult male death. According to the cancer statistics released by China in 2015, PCa accounted for about 2.4% of new cancers in men, including about 60,300 new cases and 26,600 deaths (Prendeville et al., 2017; Davidson et al., 2018). In 2018, PCa comprised about one-fifth of the total number of new cancers in American men, with a total of about 164,649 new cases and 29,430 deaths (Abouhashem and Salah, 2020). About one-third of cases experience disease recurrence, disease progression, or eventually develop into metastatic disease after initial treatment. Unfortunately, in patients with metastatic PCa, the 5-year survival rate decreased to 30%. Although there is increasing evidence that some patients with specific metastatic diseases may benefit from local treatment, such as radiotherapy and surgery (Zhou, 2018; Zupancic et al., 2020), androgen deprivation therapy (ADT) is still the main method for treatment of metastatic PCa (Lu et al., 2021). Cases with PCa and androgen deprivation treatment but still progressing will be diagnosed as castration-resistant PCa (CRPC) (Zhao et al., 2021).

Most metastatic PCa can be effectively treated with docetaxel (DTX). DTX is a drug clinically approved by the United States Food and Drug Administration for the treatment of various metastatic PCa, including androgen-independent PCa and CRPC (Hongo et al., 2021). However, as a first-line treatment for metastatic CRPC, DTX resistance is an important clinical problem. The newly developed new-generation chemotherapeutic drugs for the treatment of DTX-resistant PCa patients are often accompanied by hematotoxicity exceeding their benefits. Although DTX plays a role in the treatment of hormone-refractory PCa, DTX resistance can easily occur due to microtubule mutation and the activation of drug efflux pump after long-term treatment (Bello et al., 2021; Kobayashi et al., 2021). Therefore, studying the chemical resistance mechanism of DTX can improve the efficiency of chemotherapy. Aerobic glycolysis is mainly used by cells to provide energy and materials for their rapid growth, and glycolysis is closely related to the occurrence, development, and metastasis of malignant tumors (Shao et al., 2021). Therefore, the study of cellular glycolysis may be an important target for the treatment of malignant tumors.

MicroRNA (miRNA) is a non-coding RNA with a length of about 18.23 nt naturally generated in cells. It mainly regulates gene expression at the posttranscriptional level. It also inhibits the expression of target genes and affects the function of individual cells by binding to the 3’UTR untranslated region of its target gene messenger RNA (mRNA) (Mafi et al., 2021). However, there are only a few studies on the mechanism of drug resistance and glycolysis of miRNA in PCa. Studies have shown that miR-103a-3p plays a role in a variety of tumors (Huang et al., 2021; Li et al., 2021; Ma et al., 2021), but only a few studies in PCa exist, and its role in chemoresistance and glycolysis is unknown. Therefore, our study will clarify the genetic role of miR-103a-3p in PCa, screen and verify the target genes directly regulated by miR-103a-3p, and analyze the effects of miR-103a-3p regulating its target genes on DTX resistance and glycolysis in PCa, which will lay a certain foundation for the clinical diagnosis and treatment of miR-103a-3p in PCa.

## Materials and Methods

### Material Science

For cells and plasmids, the PCa cell PC-3 was provided by the Cell Bank of the Typical Culture Preservation Center of the Chinese Academy of Sciences. Normal prostate epithelial cell RWPE-1 was provided by the American Type Culture Collection (ATCC). Plasmids overexpressing miR-103a-3p (miR-103a-3p mimic) and TRIM66 interference (si-TRIM66) and their negative controls (mimic-NC and si-NC, respectively) were designed and constructed by the Shanghai Jima Company (Shanghai, China). The cell culture medium (RPMI 1640), fetal bovine serum (FBS), double antibody, defined keratinocyte serum-free medium (K-SFM), RNA extraction kit, MTT (3-[4,5-dimethylthiazol-2-yl]-2,5 diphenyl tetrazolium bromide) kit, and the protein extraction kit were all provided by Kaiji Biology Company. The glucose and lactic acid kits were provided by Lyle Biotech. Transwell cell was purchased from Beijing Mengzhuang Technology Co., Ltd. (Beijing, China) and Lipofectamine 3000 was from Life Technologies (Carlsbad, CA, USA).

## Methods

### Cell Culture

PC-3 cells were cultured in RPMI medium containing 10% FBS and 1% double antibody (100%) × penicillin streptomycin solution. Before cell subculture, cell growth was observed under an inverted microscope. Subculture was performed when the cell density reached 80%–90% (do not subculture if the cell state is poor). The normal prostatic epithelial cell RWPE-1 was subcultured in a medium containing 5 ng/ml epidermal growth factor, 50 mg/ml bovine pituitary extract, and 1 × K-SFM cell culture medium of antibiotics in 5% CO_2_ at 37°C.

### Target Gene Prediction

Based on the research strategy for miRNA, we screened *TRIM66* as the downstream target gene of miR-103a-3p by combining literature search and prediction using the biology website starBase (https://starbase.sysu.edu.cn).

### Real-Time Fluorescence Quantitative PCR

A certain amount of Trizol solution was added to PC-3 cells to extract total RNA from the cells. An enzyme-free Eppendorf (EP) tube and gun head and a high-precision pipette gun, among others, were used to extract RNA. Seventy-five percent ethanol solution [anhydrous ethanol and diethylpyrocarbonate (DEPC) water, 3:1] and isopropanol were pre-cooled in advance. The cells were transferred to the enzyme-free EP tube, an appropriate amount of chloroform was added, allowed to stand for 15 min, and then centrifuged. The following parameters were set: 4°C, 12,000 rpm, centrifugation for 20 min. The EP tube was taken out and the upper aqueous phase was sucked out. The volume of the supernatant was calculated and an equal volume of pre-cooled isopropanol was added. After shaking briefly on the oscillator, centrifugation was again done at 4°C, 12,000 rpm, for 15 min, the supernatant was discarded, 1 ml 75% ethanol solution was added, re-centrifuged, and the supernatant discarded. A white RNA precipitation can be seen at the bottom of the EP tube. Of the RNA sample, 2 µl was taken and a spectrophotometer was used to detect the optical density, OD_260/280_. A value between 1.9 and 2.0 denotes high RNA purity and can then be used for subsequent experiments. The remaining RNA was labeled and stored at −80°C and the RNA reverse transcribed into complementary DNA (cDNA). The primers were purchased from Shanghai Gemma Company (Shanghai, China) and the reverse transcription kit purchased from Thermo company. Performed according to the user guide in the fluorescence quantitative kit, U6 and GAPDH were taken as the internal parameters and the relative contents of miR-103a-3p and TRIM66 between PC-3 and RWPE-1 calculated with the 2^−ΔΔCT^ method (Wang G et al., 2021). The primer sequences are shown in Table 1.

**TABLE 1:** qRT-PCR primers

Primer sequence	Forward (5′–3′)	Reverse (5′— 3′)
miR-103a-3p	AGCAGCATTGTACAGG	CTCTACAGCTATATTGC
	GCTATG	CAGCCAC
TRIM66	GTA​TGA​CCA​GAA​GAA​GTG​TGA​G	CTG​ACA​GGT​TCA​TGG​AAG​G
U6	CTCGCTTCGGCAGCACA	AAC​GCT​TCA​CGA​ATT​TGC​GT
GAPDH	CAA​TAG​TGA​TGA​CCT​GGC​CGT	AGA​GGG​AAA​TCG​TGC​GTG​AC

### Dual Luciferase Assay

According to the binding site between miR-103a-3p and TRIM66 predicted using starBase (https://starbase.sysu.edu.cn), the wild-type TRIM66 (TRIM66-WT) plasmid and mutant TRIM66 (TRIM66-MUT) plasmid were established. The experiment was carried out with small and medium plasmid extraction kits purchased from Tiangen Biochemical Technology (Beijing, China) according to the manufacturer’s instructions and the plasmid solution collected into a centrifuge tube until further use. The state and growth of PC-3 cells were observed. Better quality cells were co-transfected with the miR-103a-3p mimic, mimic-NC, TRIM66-WT, and TRIM66-MUT in 24-well plates. The complete medium was replaced 4–6 h after transfection. The luciferase value could be detected 48 h later. The obtained value is the luciferase value of firefly. Immediately after detection, 50 µl stop solution was added, blown evenly, and the machine for detection immediately started. The obtained value is the luciferase value of Haishen. Results were obtained by dividing the luciferase value of sea kidney by the luciferase value of firefly.

### Induction of DTX-resistant Cells

DTX-resistant PCa strains were induced by gradually increasing the concentration of DTX. The initial concentration of DTX was 0.008 µg/L. After continuous culture *in vitro* for 14 months, the drug-resistant strain grew stably to a final concentration of 5 µg/L DTX. DTX-resistant cells were successfully induced.

### The Drug Resistance of DTX-resistant Cells Was Detected by MTT

PCa and DTX-resistant cells in the logarithmic growth stage were inoculated on 96-well plates with an inoculation density of 2 × 10^4^ wells. After cell adherence to the wall and growth, different concentrations of DTX were added. The final concentrations of DTX were 100, 50, 20, 10, 5, 2, and 1 µg/L. Three multiple holes were set up for each group of data. After 72 h of drug co-culture, 2 mg/ml MTT 50 µl was added to each well and placed in an incubator at 5% CO_2_ and 37°C for 4 h. The culture medium was discarded by turning the plate, 150 µl DMSO was added to each well, and then placed in an incubator at 5% CO_2_ and 37°C for 30 min. The absorbance value (OD value) of each hole was measured at 570 nm wavelength of the microplate reader, and the cell survival rate was calculated as follows: [survival rate = (OD value of experimental group/OD value of control group) × 100%]. Taking the drug concentration as the horizontal axis, a concentration utility curve was drawn to determine the half-maximal inhibitory concentration (IC_50_). The drug resistance index was calculated as follows: IR = IC_50_ of DTX-resistant cells/IC_50_ of PCa cells.

### Drawing of the Cell Growth Curve

Logarithmic PCa and DTX-resistant cells were digested by trypsin to prepare 2 × 10^4^ cells/ml suspension, which was inoculated on 24-well plates at 1 ml/well. The cells were counted on days 1, 3, 5, and 7 after inoculation, and three wells were counted each time to draw the growth curve.

### DTX-resistant Cell Transfection and Grouping

DTX-resistant cells in the logarithmic growth stage with cell density of about 70%–80% were transfected with Lipofectamine 3000; the miR-103a-3p mimic, si-TRIM66, mimic-NC, and si-NC were also transfected. The following groups were classified: PCa group (no transfection); DTX-resistant group (no transfection); DTX-resistant + mimic-NC group (DTX-resistant group transfected with the miR-103a-3p mimic, negative control); DTX-resistant + miR-103a-3p mimic group (DTX-resistant group transfected with the miR-103a-3p mimic); DTX-resistant + si-NC group (DTX-resistant group with TRIM66 interference, negative control); and DTX-resistant + si-TRIM66 group (DTX-resistant group with TRIM66 interference). Quantitative real-time PCR (qRT-PCR) was used to detect the expressions of miR-103a-3p and TRIM66 in each group.

### The Colony Forming Ability of DTX-resistant Cells Was Detected by Cell Cloning Assay

Transfected cells were cultured in a Petri dish with a density of 6 × 10^2^ cells/dish. Cell fluid exchange was performed at 3–4 days and the progress of cell mass size was observed. After the cell mass size reached 50 cells/mass, fixation and staining were performed. For cell fixation, the culture medium was sucked out of the orifice plate, PBS was added, and washing was done two or three times. After sucking out PBS, methanol was added at 500 µl and fixed at room temperature for 15 min. Methanol was then sucked out and 500 µl crystal violet was added for 20 min at room temperature. Then, crystal violet was sucked out, PBS was added, and washing was performed two or three times. Spare photos were kept for further analysis of the results. The number of colonies with more than 50 cells was directly calculated by naked eye or counted using a low-power microscope.

### The Migration Ability of DTX-resistant Cells Was Detected by Wound Healing Experiment

The transfected cells of each group were cultured in a Petri dish. When the cell growth density was fused, the cells of each group were scratched along the scale with a 10-µl sterile gun head on an ultra-clean workbench, and then the scratched off-wall floating cells were gently washed with sterile PBS. After 0-h scratch photography, 1% blood serum fresh culture medium was added. After 24 h, the scratch healing rate was calculated as the ratio of the difference between the scratched area at 0 and 24 h to the scratch area at 0 h. The scratch healing rate was calculated to understand the migration and repair of cells in each group.

### The Invasion Ability of DTX-resistant Cells Was Detected by the Transwell Assay

Cells in good growth condition were selected and placed in 24-well plates on an ultra-clean sterile worktable, 500 µl of complete culture medium was added into the wells, and then the wells placed in the Transwell chamber with matrix glue evenly spread on the upper layer. After cell digestion, the cells were resuspended in serum-free medium, the final cell density was adjusted to 1 × 10^5^/100 µl, and the cells added into the Transwell chamber. The 24-well plates were placed into the carbon dioxide cell incubator and cultured for 24 h. After cell culture in the Transwell chamber for 24 h, the chamber was taken out and the non-adherent cells in the lower layer of the chamber gently washed away with PBS solution. The cells in the upper layer of the chamber that did not migrate to the lower layer were then gently wiped off with a big cotton swab, placed into the chamber in a 24-well plate containing 500 µl methanol for 15 min, and then the cells transferred into a 24-well plate containing 500 µl crystal violet for staining for 20 min. Finally, the cells were taken out, placed in PBS solution to gently wash away the crystal violet that did not combine with cells, and the residual cells and crystal violet on the upper layer of the cell were again wiped off with a cotton swab. The cells were placed upside down on a super clean sterile workbench, the cells allowed to dry, and then the collected cells placed in a new 24-well plate for photos. Five different visual fields were randomly selected from each chamber to calculate the number of cells passing through.

### Detection of Glycolysis

After cell transfection, the culture medium was collected and the cell fragments discarded by centrifugation. The levels of glucose and lactic acid in the culture medium were calculated; together with the cell count, the background value of the culture medium was subtracted from the obtained glucose and lactic acid contents and divided by the number of corresponding cells to obtain the sugar consumption and lactic acid production of each cell. The results were expressed in relative multiples, with the cells in the control group used as the standard.

### Statistical Methods

SPSS 19.0 statistical software was used for analysis of the data. Measurement data were expressed as the mean ± SD, and measurement data subject to normal distribution were compared between two groups using *t*-test and paired *t*-test. A *p* < 0.05 indicated that the difference was statistically significant.

## Results

### Expressions of miR-103a-3p and TRIM66 in PCa Cells

**FIGURE 1:**
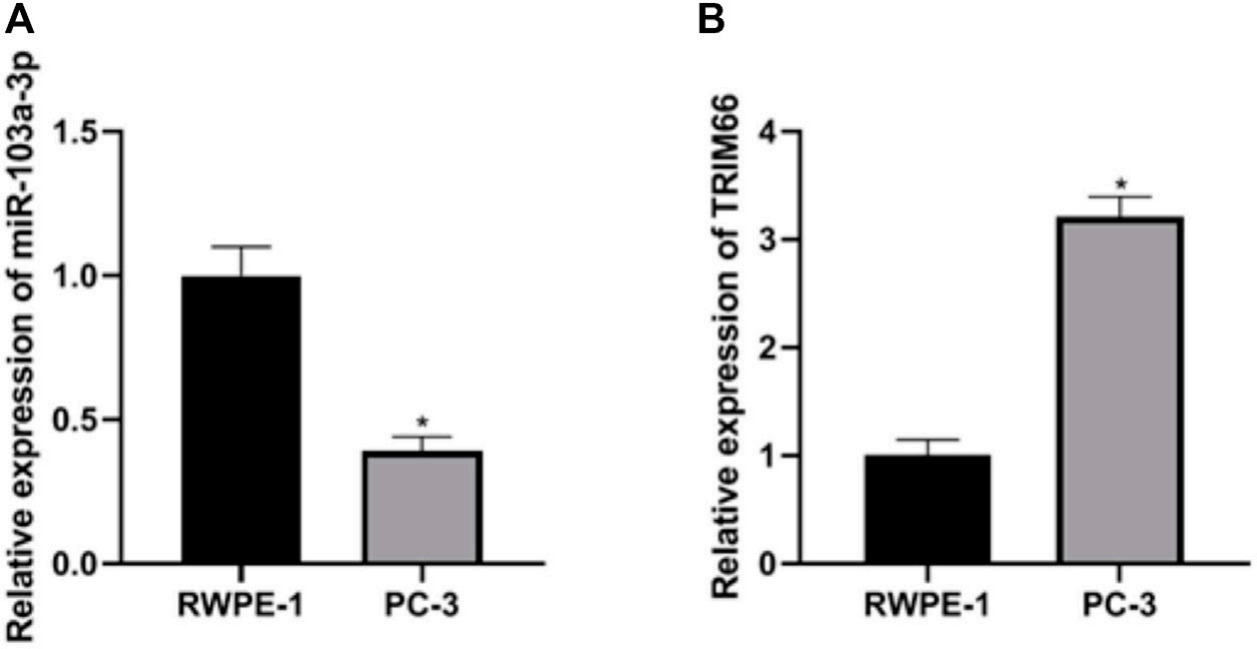
Expressions of miR-103a-3p and TRIM66 in prostate cancer (PCa) cells. *p < 0.05 (compared with RWPE-1).

The expressions of miR-103a-3p and TRIM66 in PCa cells were examined in the human normal prostate epithelial cells RWPE-1 and the PCa cell PC-3 using qRT-PCR. The results showed that, compared with RWPE-1 cells, the expression of miR-103a-3p (0.39 ± 0.05) was lower and that of TRIM66 (3.21 ± 0.19) was higher in the PCa cell PC-3 (*p* < 0.05), as shown in Figures 1A, B. This suggests that miR-103a-3p and TRIM66 may be involved in the progression of PCa.

### Relationship Between miR-103a-3p and TRIM66

**FIGURE 2:**
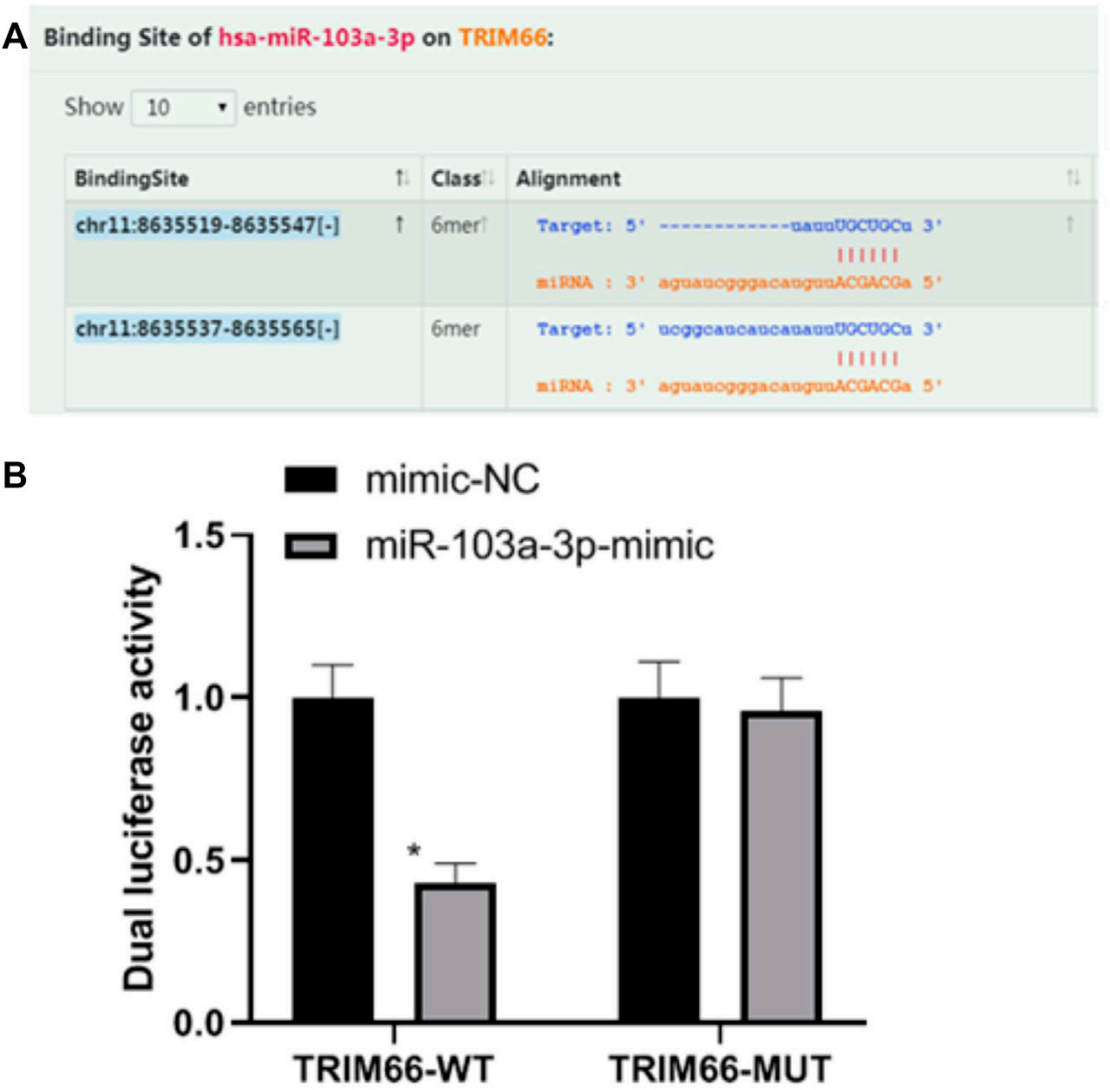
Relationship between miR-103a-3p and TRIM66. *p < 0.05 (compared with mimic-NC). Source for **(A):** https://starbase.sysu.edu.cn.

Based on the research strategy for miRNA, we screened *TRIM66* as the downstream target gene of miR-103a-3p by combining literature search and prediction on the biology website starBase (https://starbase.sysu.edu.cn). In order to verify the relationship between miR-103a-3p and TRIM66, we performed a dual luciferase reporter assay. The results showed that the luciferase activity of TRIM66-WT transfected with the miR-103a-3p mimic decreased (0.43 ± 0.06, *p* < 0.05), while that of TRIM66-MUT transfected with the miR-103a-3p mimic did not change significantly (0.96 ± 0.10, *p* > 0.05), as shown in Figures 2A, B. This suggests that there is a targeting relationship between miR-103a-3p and TRIM66.

### Drug Resistance Characteristics and Growth Curve of DTX-resistant Cells

**FIGURE 3:**
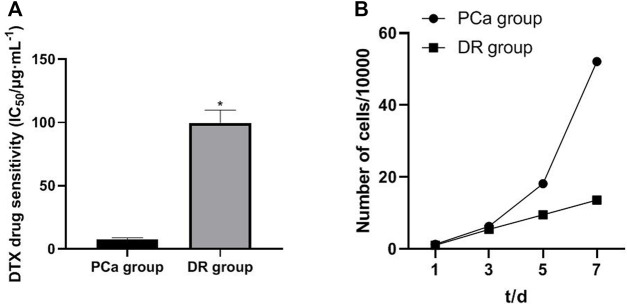
Drug resistance characteristics and growth curve of DTX-resistant cells. *p < 0.05 [compared with the prostate cancer (PCa) group].

In order to investigate the relationship between miR-103a-3p, TRIM66, and DTX resistance in PCa, DTX-resistant cells were established. By detecting the drug resistance of DTX-resistant cells, it was found that, compared with cells in the PCa group, those in the DTX-resistant group showed increased resistance to DTX. The drug resistance index was 13.33, and the doubling time of the DTX-resistant group cells was significantly longer than that of the PCa group cells (*p* < 0.05), as shown in Figures 3A, B. As for the relationship between the tumor doubling time and the efficacy of chemotherapy, it is considered that the shorter the tumor doubling time, the more sensitive to chemotherapy drugs and the better the efficacy. On the contrary, the longer the doubling time of the tumor, the lower the sensitivity to chemotherapeutic drugs (Han et al., 2010). Therefore, the results suggest that DTX-resistant cells were successfully induced.

### Expressions of miR-103a-3p and TRIM66 in DTX-resistant Cells

**FIGURE 4:**
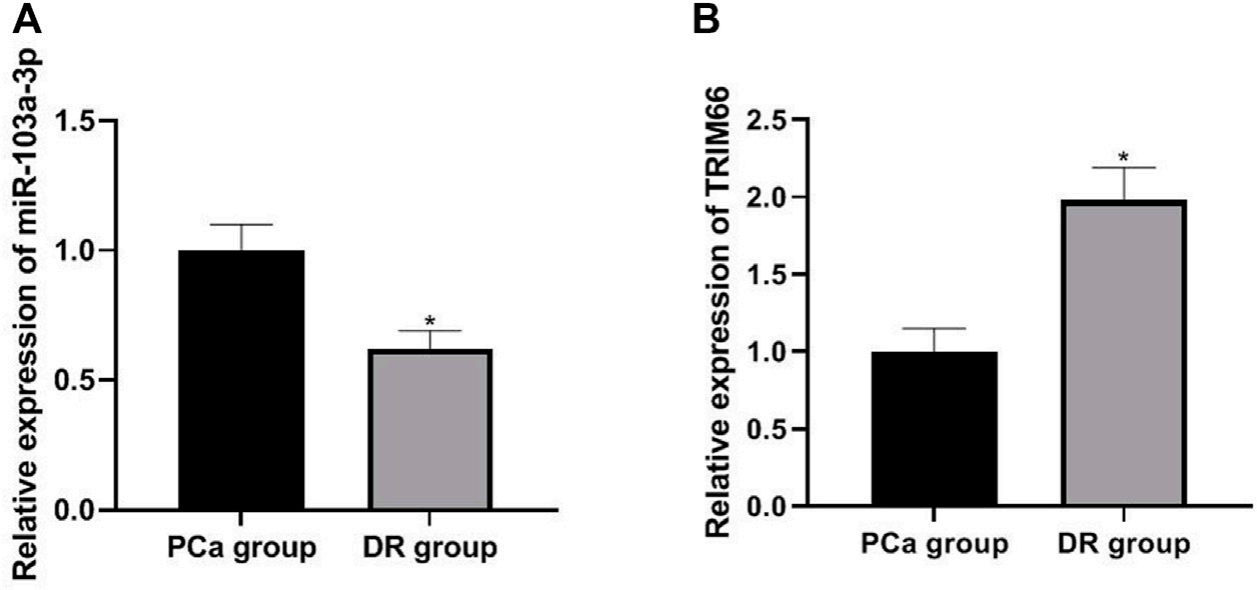
Expressions of miR-103a-3p and TRIM66 in DTX-resistant cells.

The expressions of miR-103a-3p and TRIM66 in DTX-resistant cells were detected in the PCa group and DTX-resistant group cells by qRT PCR. The results showed that the expression of miR-103a-3p (0.62 ± 0.07) was lower and that of TRIM66 (1.98 ± 0.21) was higher in the DTX-resistant group than in the PCa group (*p* < 0.05), as shown in Figures 4A, B. This suggests that miR-103a-3p and TRIM66 may be involved in the occurrence of DTX resistance.

### Expressions of miR-103a-3p and TRIM66 in DTX-resistant Cells After Transfection

**FIGURE 5:**
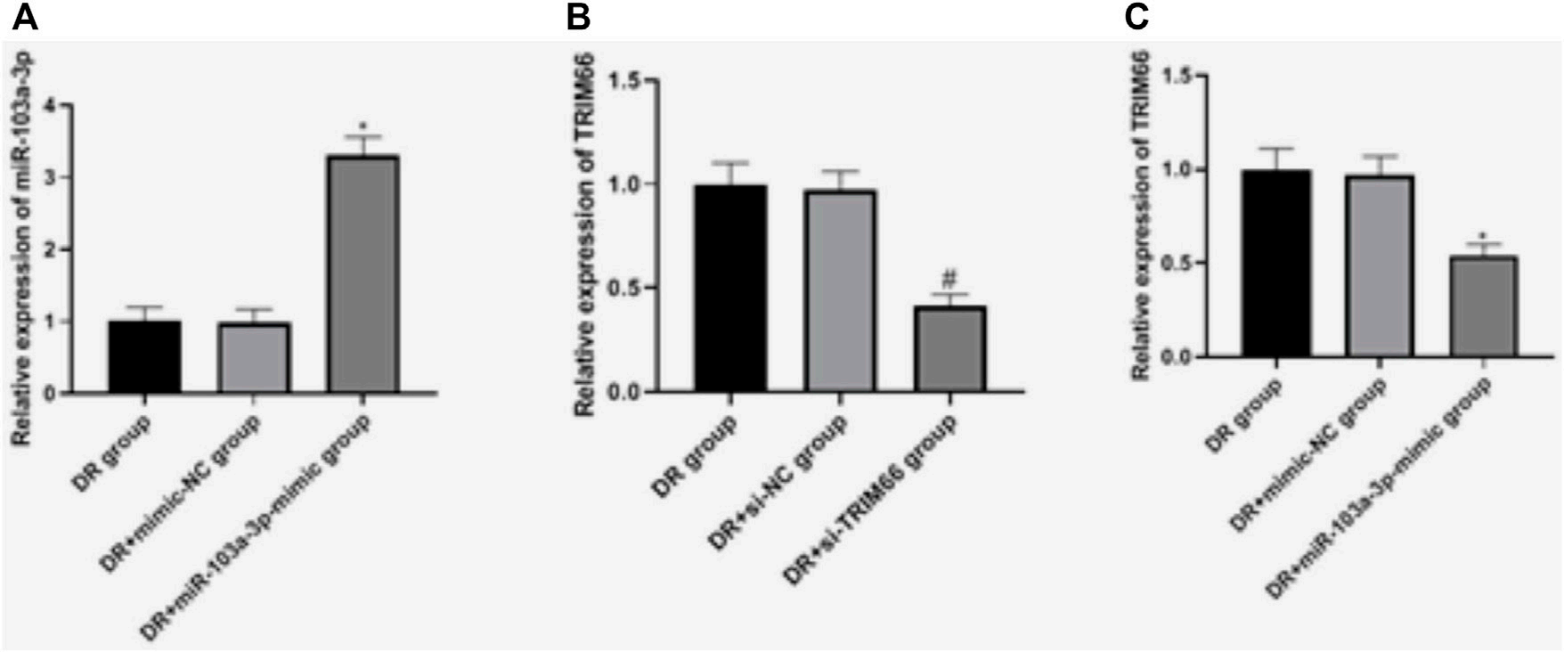
Expressions of miR-103a-3p and TRIM66 in DTX-resistant cells after transfection. *p < 0.05 (compared with the DTX-resistant + mimic-NC group); ^#^ *p* < 0.05 (compared with the DTX-resistant + si-NC group).

In order to investigate the effects of miR-103a-3p and TRIM66 on DTX resistance, we regulated their expressions in DTX-resistant cells. qRT-PCR showed that there was no significant difference in the expressions of miR-103a-3p between the DTX-resistant group and the DTX-resistant + mimic-NC group (*p* > 0.05). After overexpression of miR-103a-3p, it (3.31 ± 0.25) increased significantly in the DTX-resistant + miR-103a-3p mimic group (*p* < 0.05). There was no significant difference in the expression of TRIM66 between the DTX-resistant group and DTX-resistant + si-NC group (*p* > 0.05). After interference with TRIM66, the expression of TRIM66 (0.41 ± 0.06) decreased significantly in the DTX-resistant + si-TRIM66 group (*p* < 0.05), as shown in Figures 5A, B. This suggests that the transfection regulation of DTX-resistant cells was successful. We also found that TRIM66 (0.54 ± 0.06) decreased in the DTX-resistant + miR-103a-3p mimic group compared with the DTX-resistant + mimic-NC group (*p* < 0.05; Figure 5C). The binding sites and targeting relationship between miR-103a-3p and TRIM66 indicated that both can target and regulate the expression of TRIM66.

### Effects of the Upregulation of miR-103a-3p and Interference With TRIM66 on the Colony Formation of DTX-resistant Cells

**FIGURE 6:**
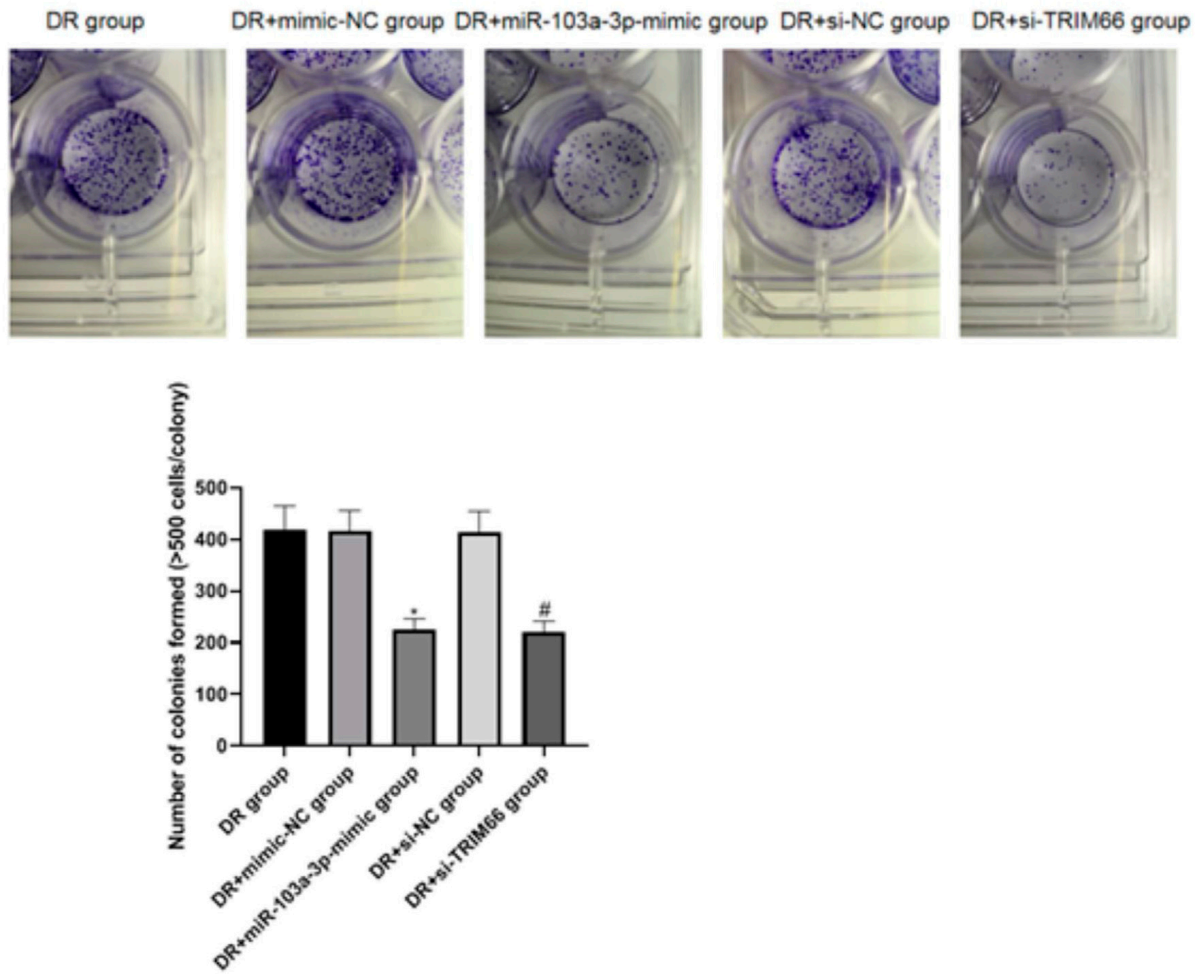
Effect of the upregulation of miR-103a-3p and interference with TRIM66 on the colony formation of DTX-resistant cells. *p < 0.05 (compared with the DTX-resistant + mimic-NC group); ^#^ *p* < 0.05 (compared with the DTX-resistant + si-NC group).

The cell cloning test was used to examine the effect of the upregulation of miR-103a-3p and interference with TRIM66 on the colony formation of DTX-resistant cells. The results showed that there was no significant difference in the number of cell colonies between the DTX-resistant group, DTX-resistant + mimic-NC group, and the DTX-resistant + si-NC group (*p* > 0.05). After overexpression of miR-103a-3p and interference with TRIM66, the colony formation of the DTX-resistant + miR-103a-3p-mimic group (225.17 ± 21.14) and the DTX-resistant + si-TRIM66 group (220.84 ± 20.68) decreased relatively (*p* < 0.05; Figure 6). This suggests that the upregulation of miR-103a-3p and interference with TRIM66 can inhibit the colony formation of DTX-resistant cells.

### Effects of the Upregulation of miR-103a-3p and Interference With TRIM66 on DTX-resistant Cell Metastasis

**FIGURE 7:**
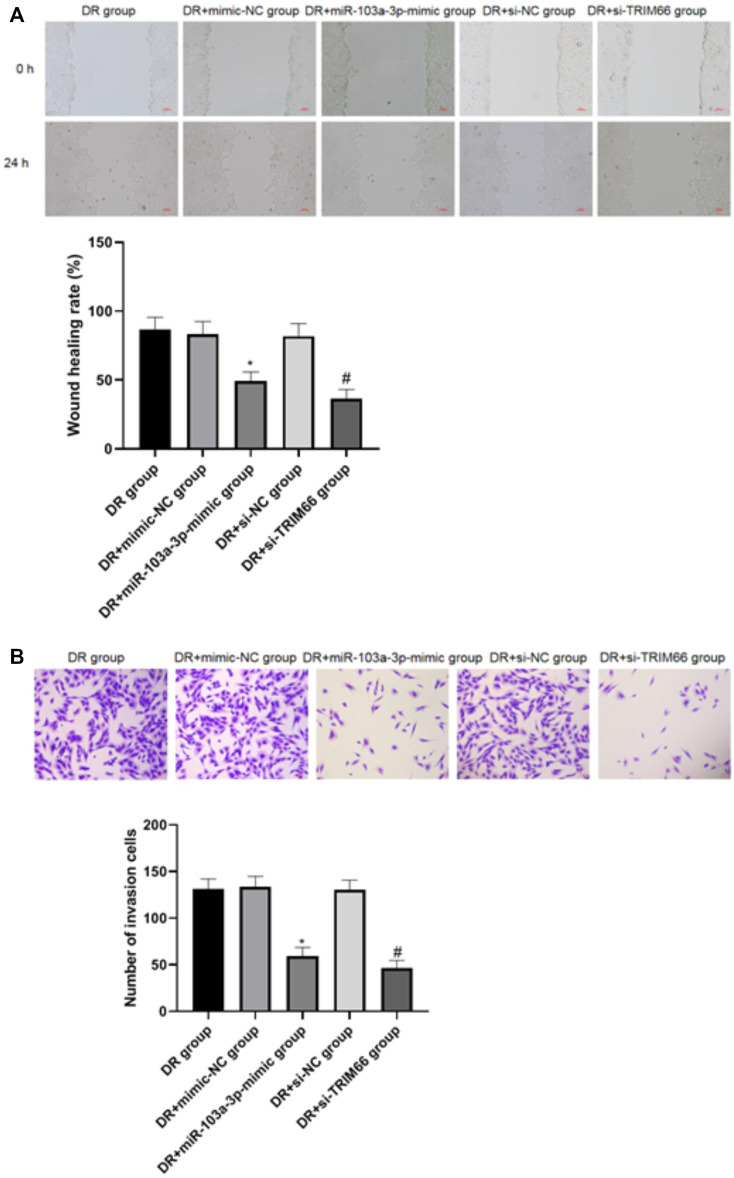
Effect of the upregulation of miR-103a-3p and interference with TRIM66 on DTX-resistant cell metastasis (×100). *p < 0.05 (compared with the DTX-resistant + mimic NC group); ^#^ *p* < 0.05 (compared with the DTX-resistant + si-NC group).

The wound healing assay and Transwell test were utilized to investigate the effects of the upregulation of miR-103a-3p and interference with TRIM66 on DTX-resistant cell metastasis. The results showed that there was no significant difference in cell migration and the number of invasive cells between the DTX-resistant group, DTX-resistant + mimic-NC group, and the DTX-resistant + si-NC group (*p* > 0.05). After overexpression of miR-103a-3p and interference with TRIM66, the cell migration ability and the number of invasive cells decreased significantly in the DTX-resistant + miR-103a-3p mimic group (49.38 ± 6.48 and 59.46 ± 9.22, respectively) and the DTX-resistant + si-TRIM66 group (36.49 ± 6.59 and 46.31 ± 8.34, respectively, *p* < 0.05), as shown in Figures 7A, B. This suggests that the upregulation of miR-103a-3p and interference with TRIM66 can inhibit DTX-resistant cell metastasis.

### Effects of miR-103a-3p and TRIM66 on the Glycolysis of DTX-resistantDTX-resistant Cells

**FIGURE 8:**
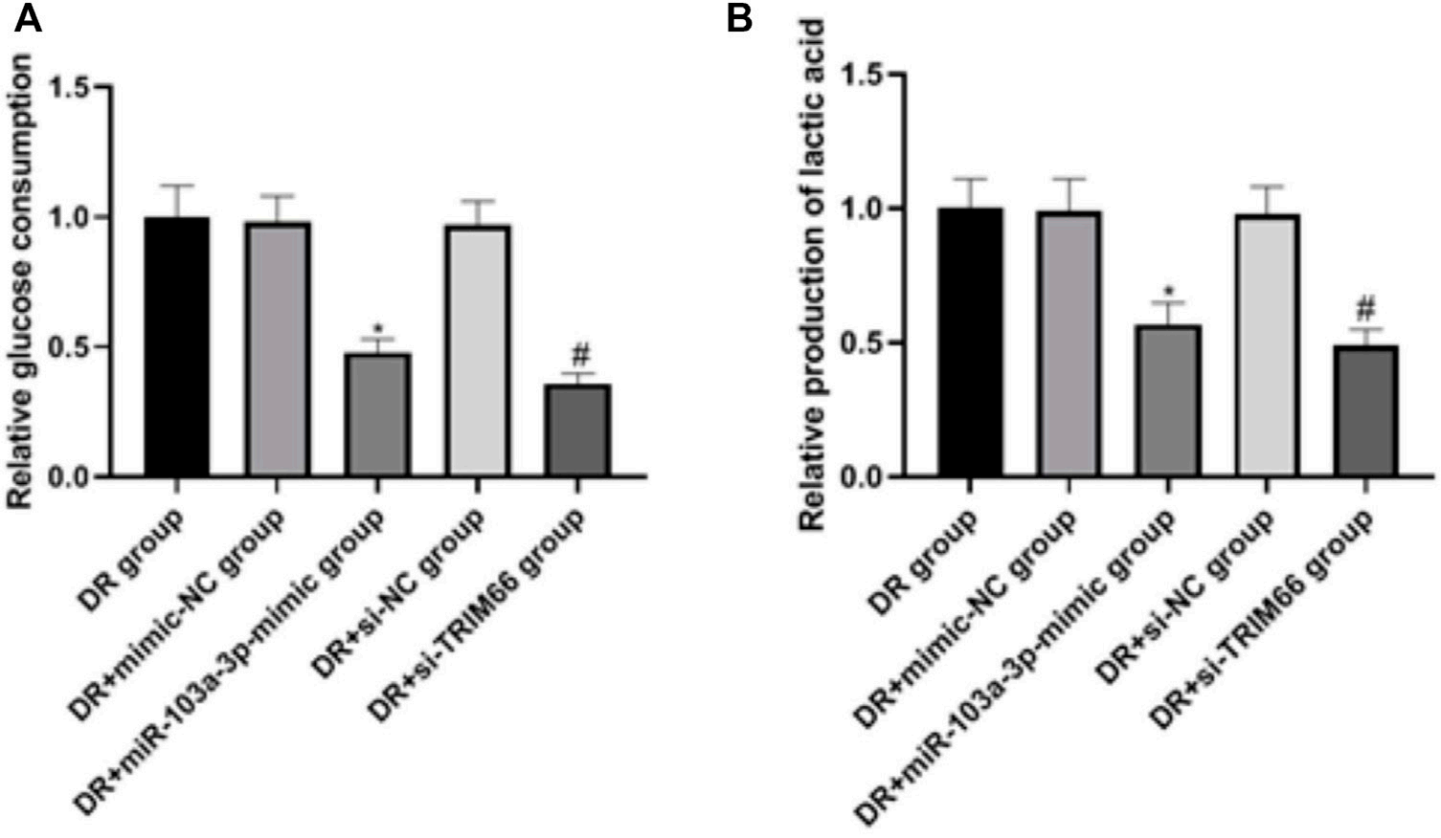
Effect of the upregulation of miR-103a-3p and interference with TRIM66 on the glycolysis of DTX-resistant cells.

In order to examine the effects of the upregulation of miR-103a-3p and interference with TRIM66 on the glycolysis of DTX-resistant cells, we used a glycolysis kit for related detection. The results showed that there was no significant difference in glucose consumption and lactate production between the DTX-resistant group, the DTX-resistant + mimic-NC group, and the DTX-resistant + si-NC group (*p* > 0.05). After overexpression of miR-103a-3p and interference with TRIM66, glucose consumption and lactate production decreased in the DTX-resistant + miR-103a-3p mimic group (0.48 ± 0.05 and 0.57 ± 0.08, respectively) and the DTX-resistant + si-TRIM66 group (0.36 ± 0.04 and 0.49 ± 0.06, respectively, *p* < 0.05), as shown in Figures 8A, B. This suggests that the upregulation of miR-103a-3p and interference with TRIM66 can inhibit the glycolysis of DTX-resistant cells.

## Discussion

PCa is a common complex disease with multiple etiologies. It is a common tumor threatening men’s health. In China, with the aging of the population, the incidence rate of PCa has been increasing year by year. It has become one of the major threats to the health of Chinese men (Raittinen et al., 2020). In recent years, with the discovery of miRNA, there have been great changes in the classical molecular biology theory. People now have a new understanding of the mechanisms of occurrence and development of malignant tumors. In this process, miRNA has brought a new dawn to PCa research with its important diagnostic and therapeutic value.

Mature miRNA binds to the target gene mRNA through nucleic acid sequence complementarity in order to inhibit the translation of the target gene mRNA or degrade it. Its function involves cell differentiation, proliferation, apoptosis, invasion, and metastasis (Qiu et al., 2021). Some scholars have studied the distribution and biological function of miRNA in mammals and found that it is involved in almost all life processes of organisms, including development events, epidermal morphogenesis, cell differentiation, enzyme activity, hormone secretion, and other regulation processes (Corrêa et al., 2021). Recent studies have shown that tumor tissues have different miRNA expression profiles compared with normal tissues. Comparison and analysis of the abnormal expression of miRNA can help to better understand the diagnosis, treatment, and prognosis of tumors. Therefore, the study of abnormal miRNA expression in PCa can help to discover important miRNA molecules, explain the mechanisms of occurrence and development of PCa, and explore its potential as a therapeutic target for new drugs.

Many studies have reported that miRNAs can target and regulate the expressions of a variety of proto-oncogenes in PCa so as to inhibit its progression, including miR-200b, miR-145, miR-224, and miR-218 (Gurbuz et al., 2021; Zabegina et al., 2021; Chen et al., 2018; Tian et al., 2020). Recent reports have indicated that miR-103 inhibits tumor cell proliferation in PCa by targeting programmed cell death protein 10 (PDCD10), while miR-103a-3p is also low in bladder cancer (Fu et al., 2016; Zhong et al., 2016). On the contrary, other studies have shown that miR-103a-3p is the proto-oncogene of many cancers, which is higher in gastric cancer, breast cancer, and ovarian cancer (Chang et al., 2016; Niu et al., 2019; Wang et al., 2021). In addition, studies have shown that miR-103a-3p is a prognostic biomarker for colon cancer and pleural mesothelioma (Caritg et al., 2016; Cavalleri et al., 2017). These often contrasting results show that the gene fDunction effects of miR-103a-3p in different cancers are usually different. Many reports have found and defined the expression profiles of disease-specific miRNAs in different tumors.

miR-103a and miR-103 are highly homologous, while miR-103a-3p is processed from the 3′-end arm of the miR-103a precursor. Our study found that the relative expression of miR-103a-3p in PCa cells was lower than that in normal prostate cells. This indicates that miR-103a-3p may be involved in the progression of PCa. Through literature search and the starBase website (https://starbase.sysu.edu.cn), we screened the target regulated by miR-103a-3p. We found that TRIM66 was highly expressed in PCa cells. This suggests that TRIM66 may be a pro-oncogene that promotes the malignant progression of PCa. In recent years, with further studies on the mechanism of chemotherapeutic drugs, chemotherapy has become one of the main methods for the clinical treatment of PCa. DTX is a taxane drug. As a first-line chemotherapy drug for the treatment of metastatic castration-resistant PCa, DTX has good clinical efficacy (Wang et al., 2020). However, after DTX treatment, about 95% of patients develop DTX resistance. Chemoresistance is inevitable in tumors that involves a variety of mechanisms, such as reducing the drug concentration of cells, increasing the cell metabolism of drug detoxification proteins, activating the survival signaling pathway, and inhibiting apoptosis (Bodzioch et al., 2021). Therefore, it is of great significance to discover new molecular targets and possible mechanisms of DTX resistance.

In further experimental studies, we established DTX-resistant cells and analyzed the low expression of miR-103a-3p and the high expression of TRIM66 in these cells, the results of which suggest that miR-103a-3p and TRIM66 may be involved in the DTX resistance of PCa. The overexpression of miR-103a-3p was found to inhibit the colony formation, migration, and invasion of DTX-resistantD cells, showing that miR-103a-3p may play the role of a tumor suppressor gene in PCa and reduce DTX resistance. It was found that the expression of TRIM66 in DTX-resistant cells decreased after the overexpression of miR-103a-3p. In addition, after interference with TRIM66, we found that TRIM66 could also inhibit DTX-resistant cells. Previous studies have shown that TRIM66 has an effect on the response to DTX resistance, which is consistent with our results that interference with TRIM66 can consistently inhibit DTX-resistant cell colony formation, migration, and invasion and that miR-103a-3p may inhibit DTX resistance and the biological behavior of cells by targeting TRIM66.

The energy metabolism of malignant tumor cells is also one of the preface and hot spots of current research, but there are still several unexplained mechanisms. Exploring new molecular targets and signal pathways is of great significance for tumor prevention and treatment. Malignant tumor cells mainly use aerobic glycolysis instead of oxidative phosphorylation of normal tissues for glucose metabolism (Warburg effect, discovered by Nobel laureate Warburg in 1920) (Guo et al., 2021). Compared with oxidative phosphorylation, glycolysis is a relatively low-productivity and low-efficiency process, but tumor cells can still quickly obtain energy ATP and growth material to supply their vigorous growth needs (Benny et al., 2020). In our study, it was also found that, after overexpression of miR-103a-3p and interference with TRIM66, the glucose consumption and lactate production of DTX-resistant cells were significantly reduced, significantly inhibiting the glycolytic flow of cells, which would slow down the growth of cancer cells and inhibit tumor.

## Conclusion

In conclusion, the expression of miR-103a-3p was downregulated and that of TRIM66 was upregulated in the malignant progression of PCa, and its expression was more obvious in DTX resistance. Upregulation of miR-103a-3p and interference with TRIM66 can inhibit DTX resistance and the glycolysis of PCa cells. Targeting TRIM66 may provide potential application value in molecular therapy for PCa.

## Data Availability

The datasets presented in this study can be found in online repositories. The names of the repository/repositories and accession number(s) can be found in the article/Supplementary Material.
