# Supplementary figures and images for: Effects of miR-103a-3p Targeted Regulation of TRIM66 Axis on Docetaxel Resistance and Glycolysis in Prostate Cancer Cells
Source: Front Genet. 2022 Feb 8;12:813793. doi: 10.3389/fgene.2021.813793 (PMC8861206; doi:10.3389/fgene.2021.813793)

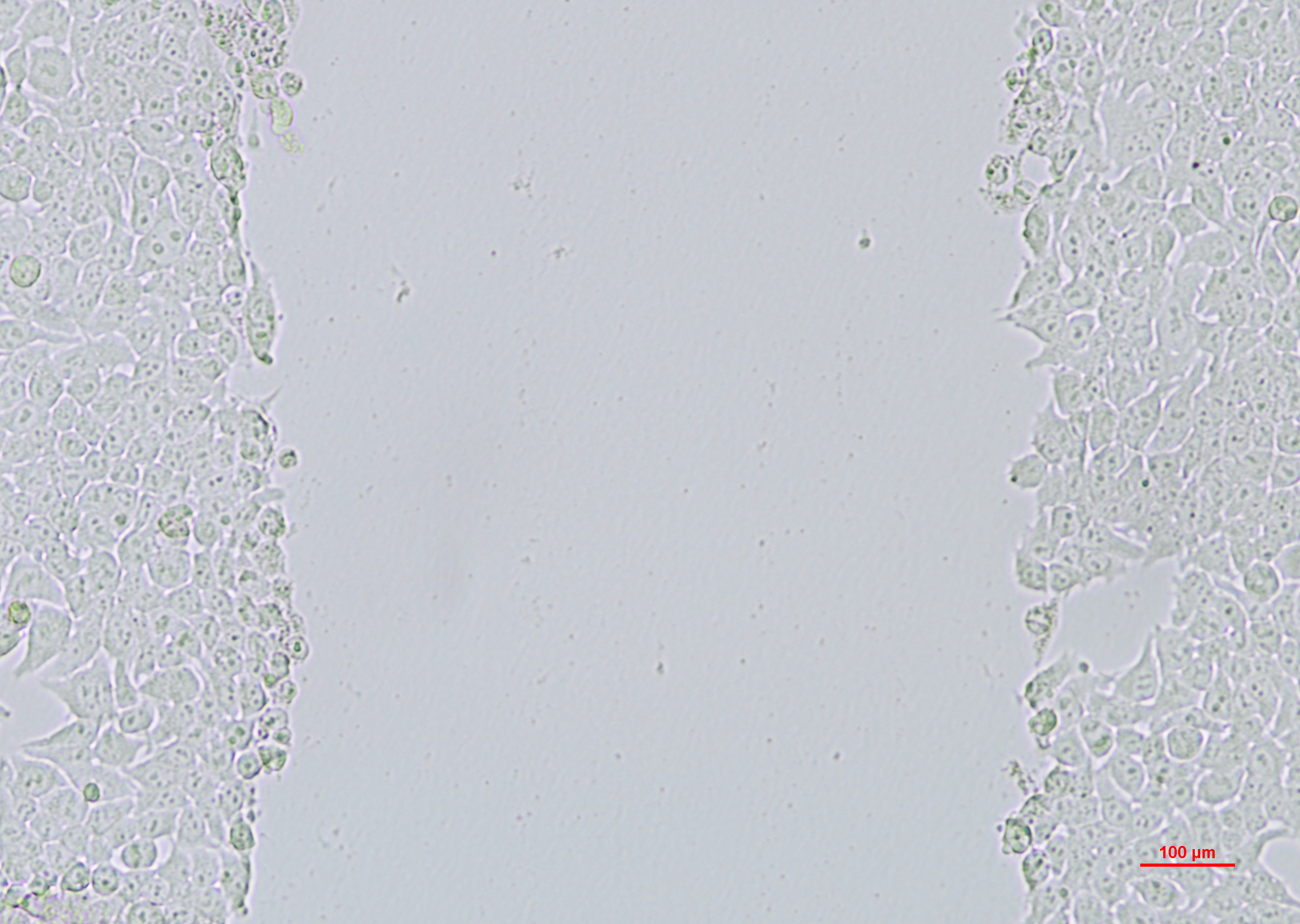

Supplement: Supplementary file 1 [file Image6.TIF]

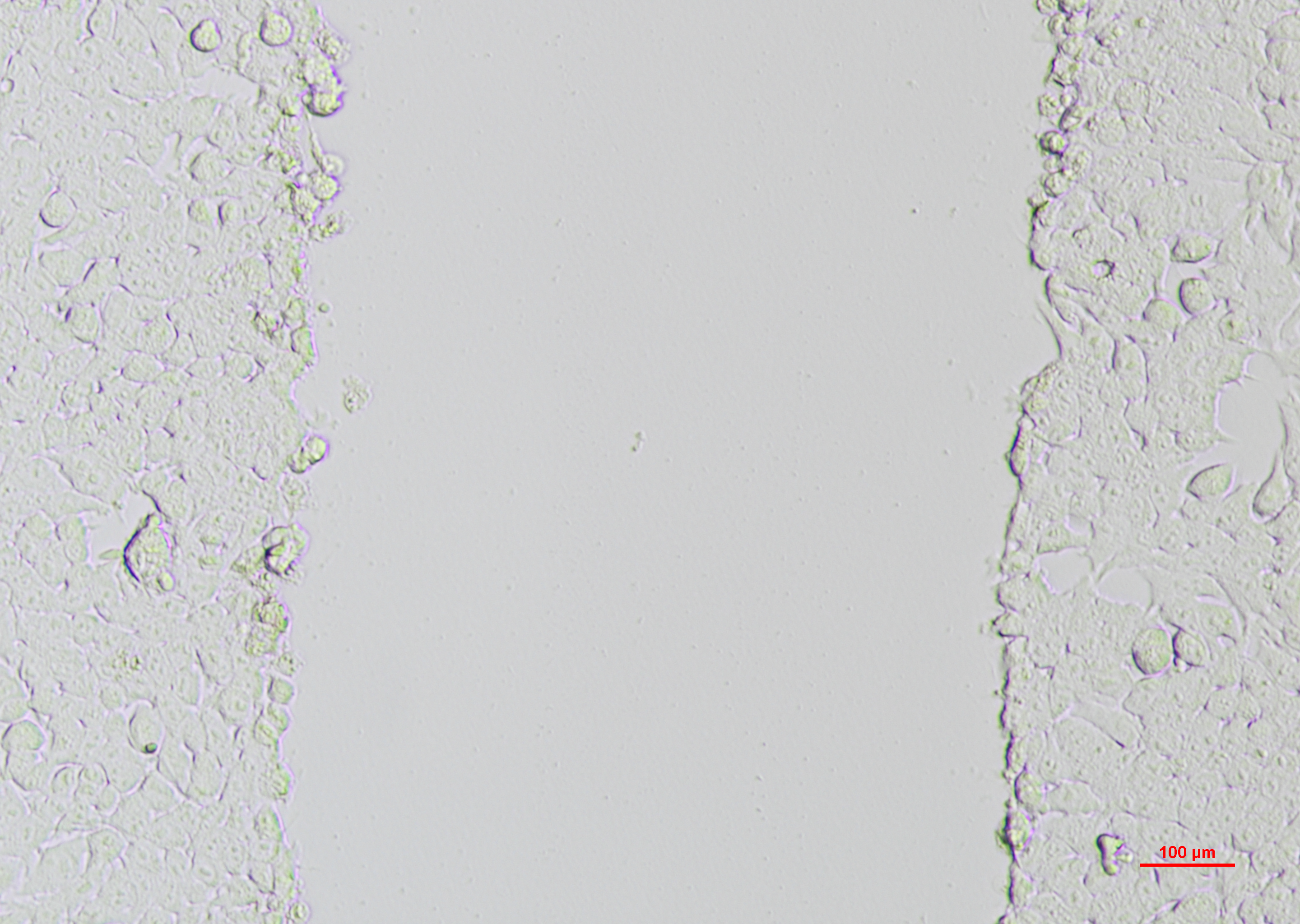

Supplement: Supplementary file 2 [file Image14.TIF]

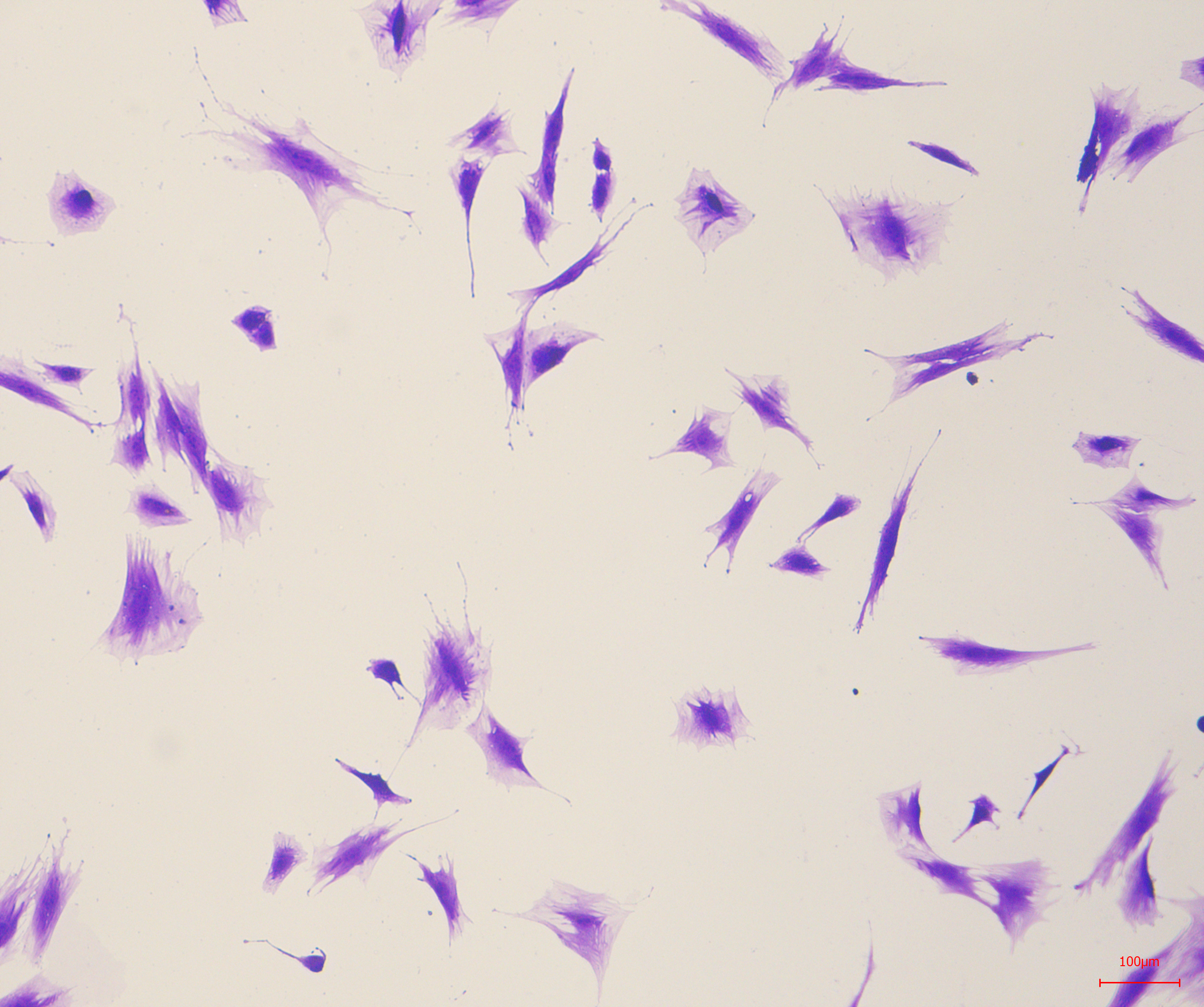

Supplement: Supplementary file 3 [file Image3.TIF]

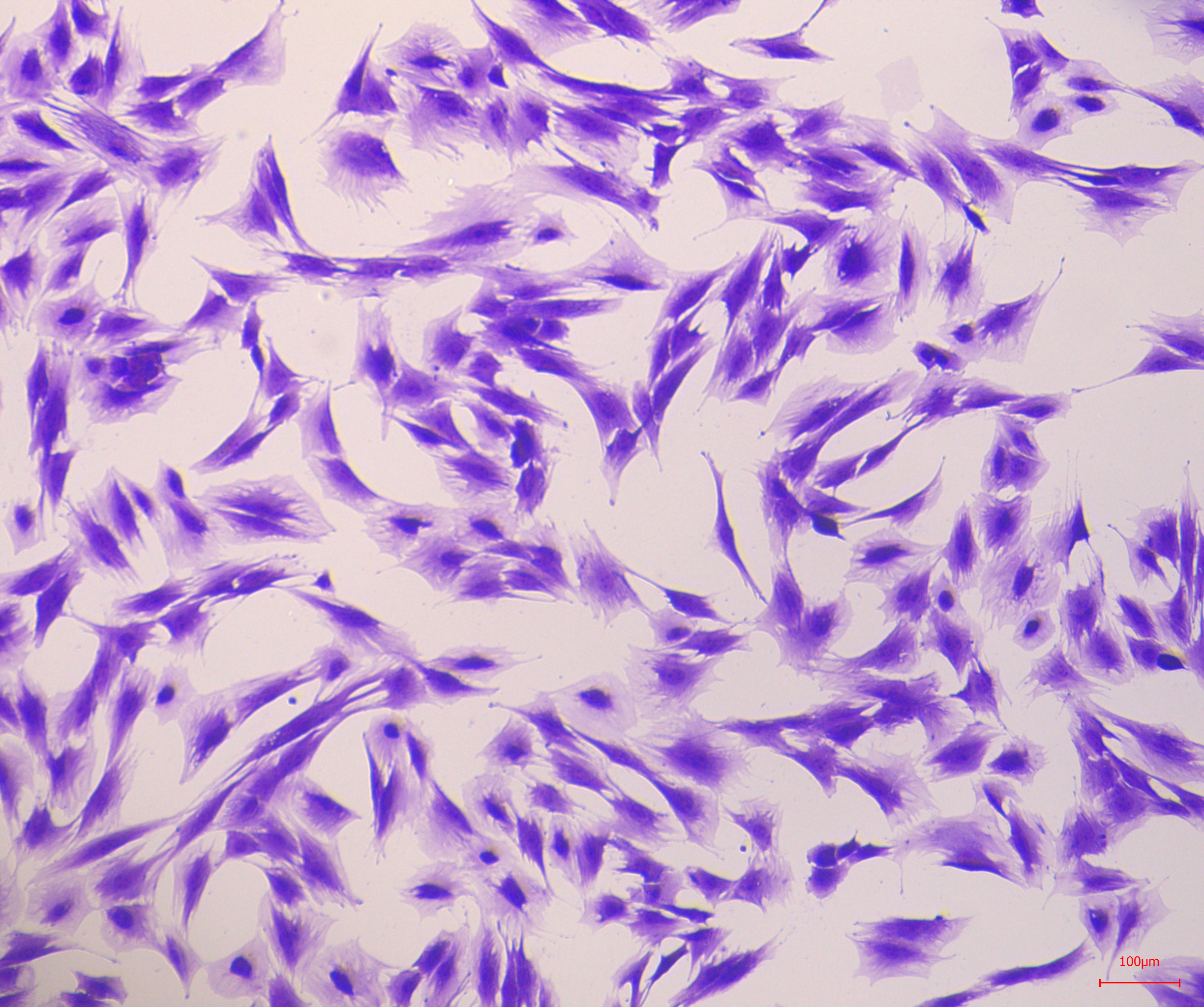

Supplement: Supplementary file 4 [file Image4.TIF]

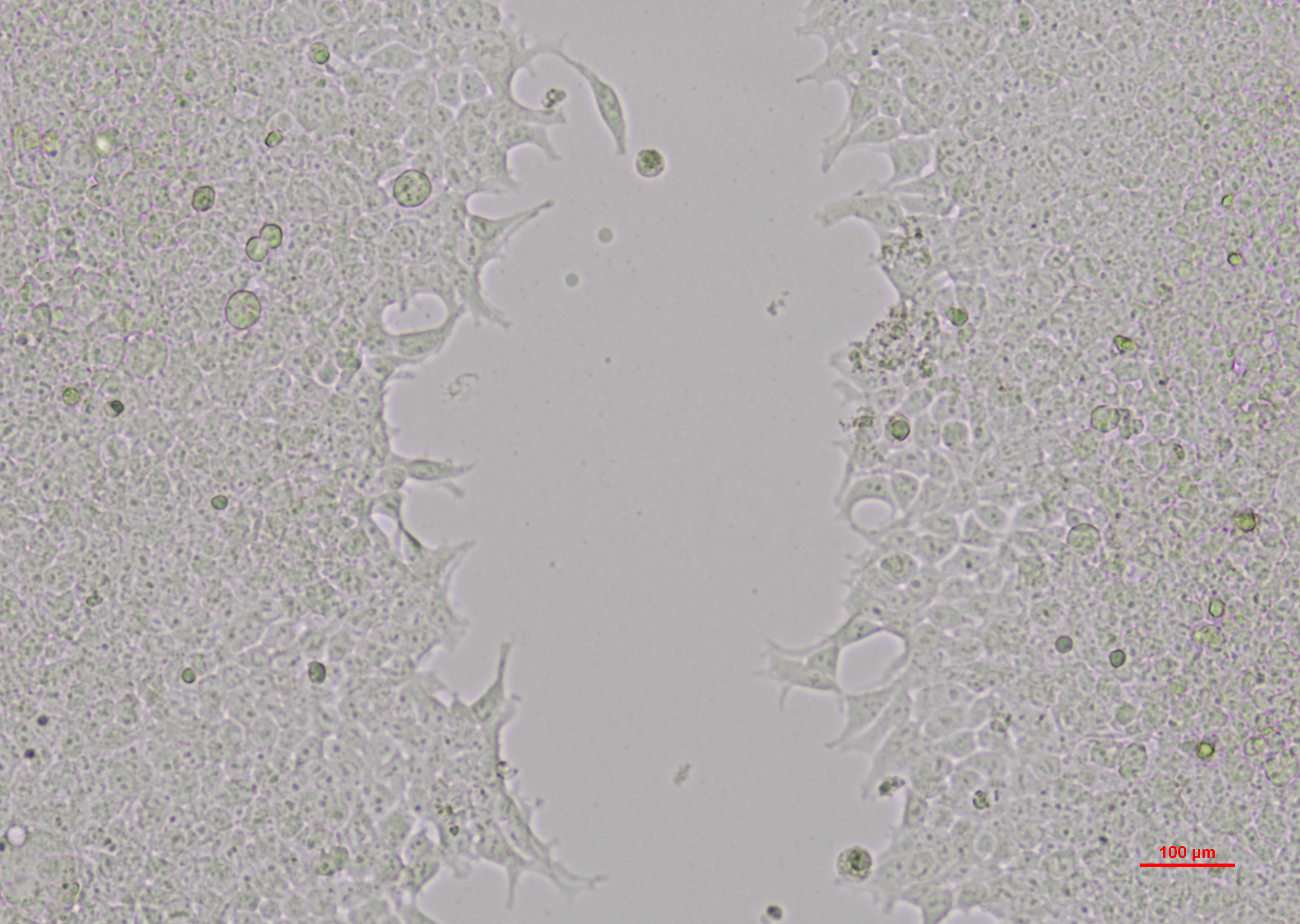

Supplement: Supplementary file 5 [file Image9.TIF]

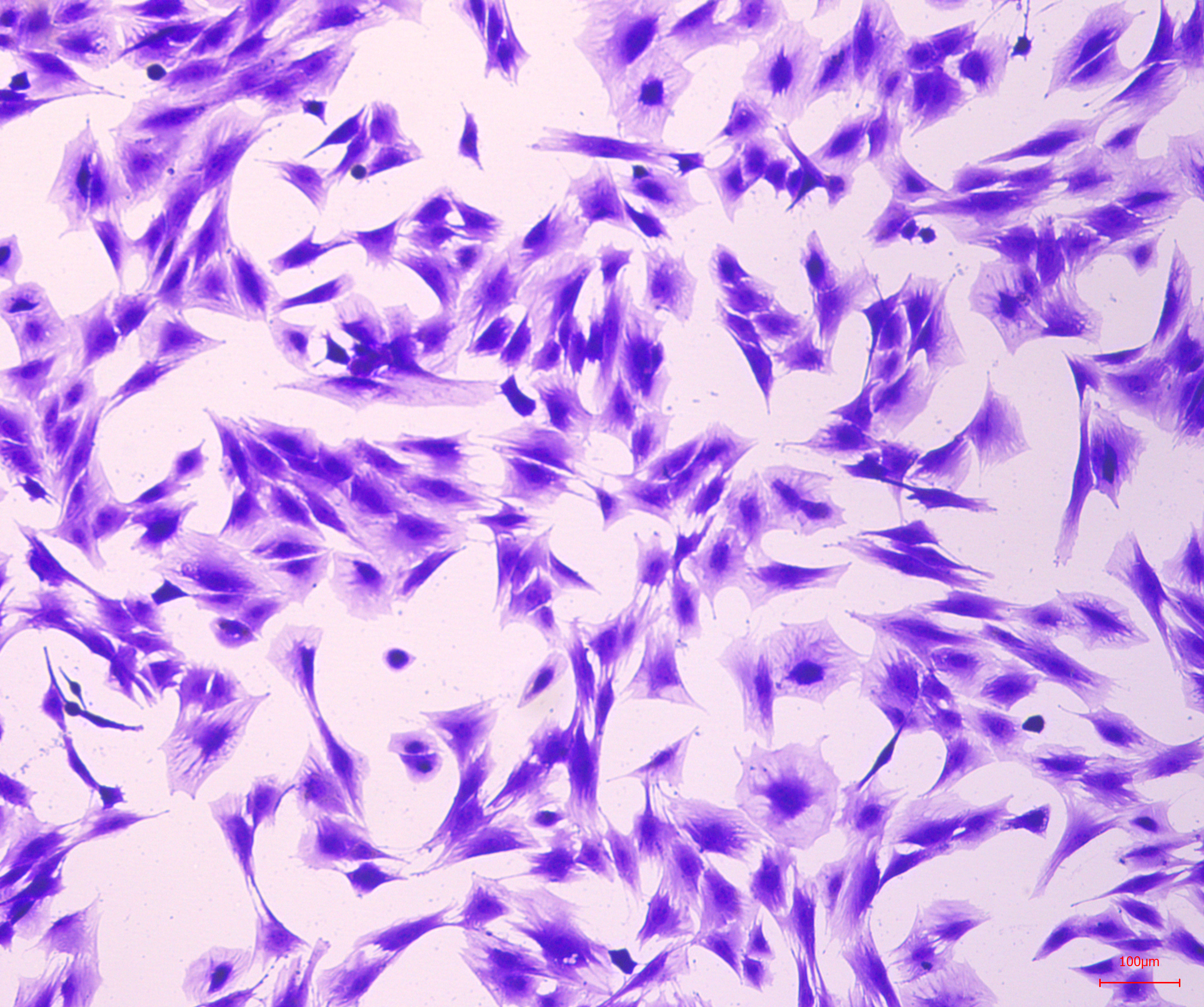

Supplement: Supplementary file 7 [file Image2.TIF]

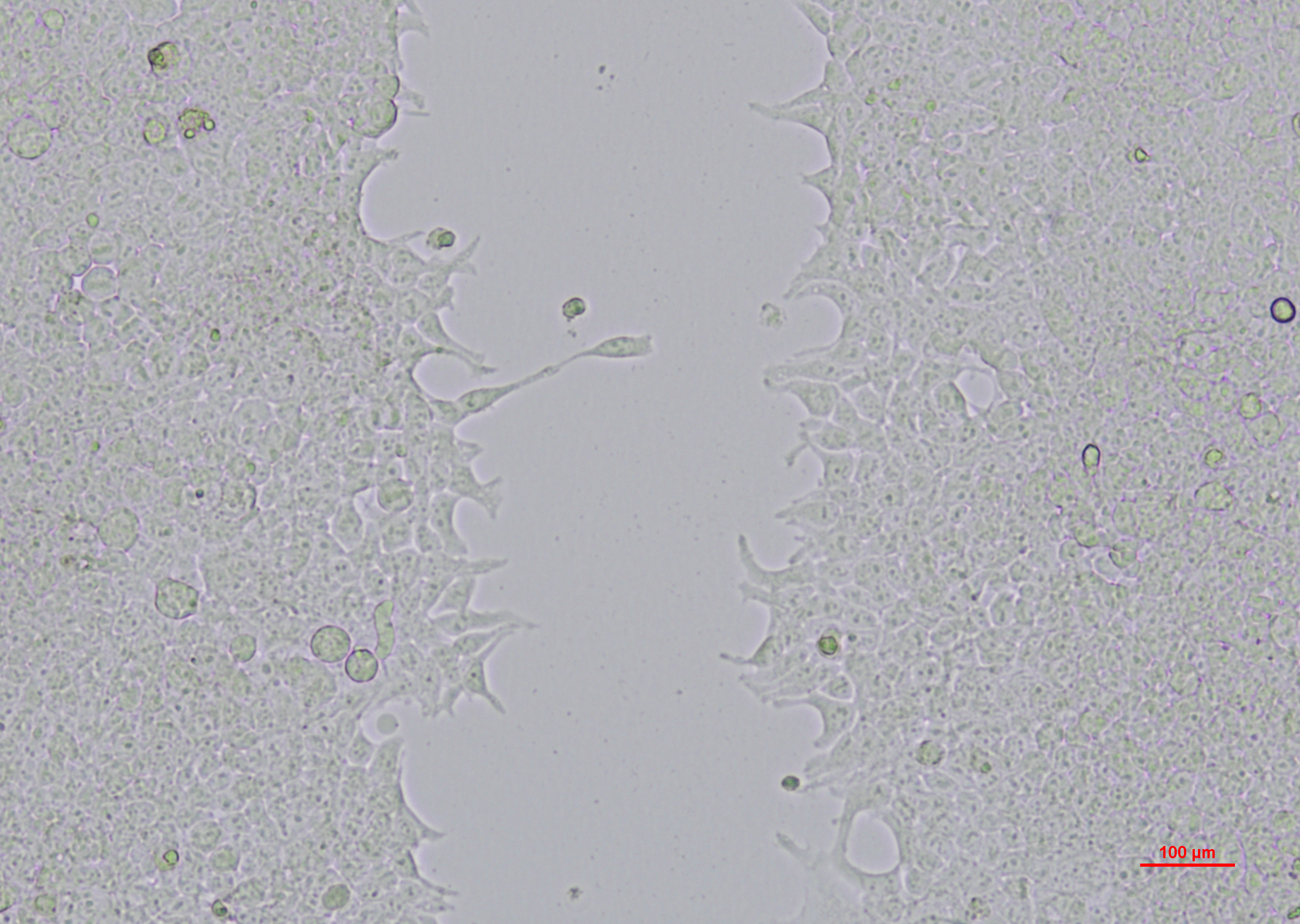

Supplement: Supplementary file 8 [file Image13.TIF]

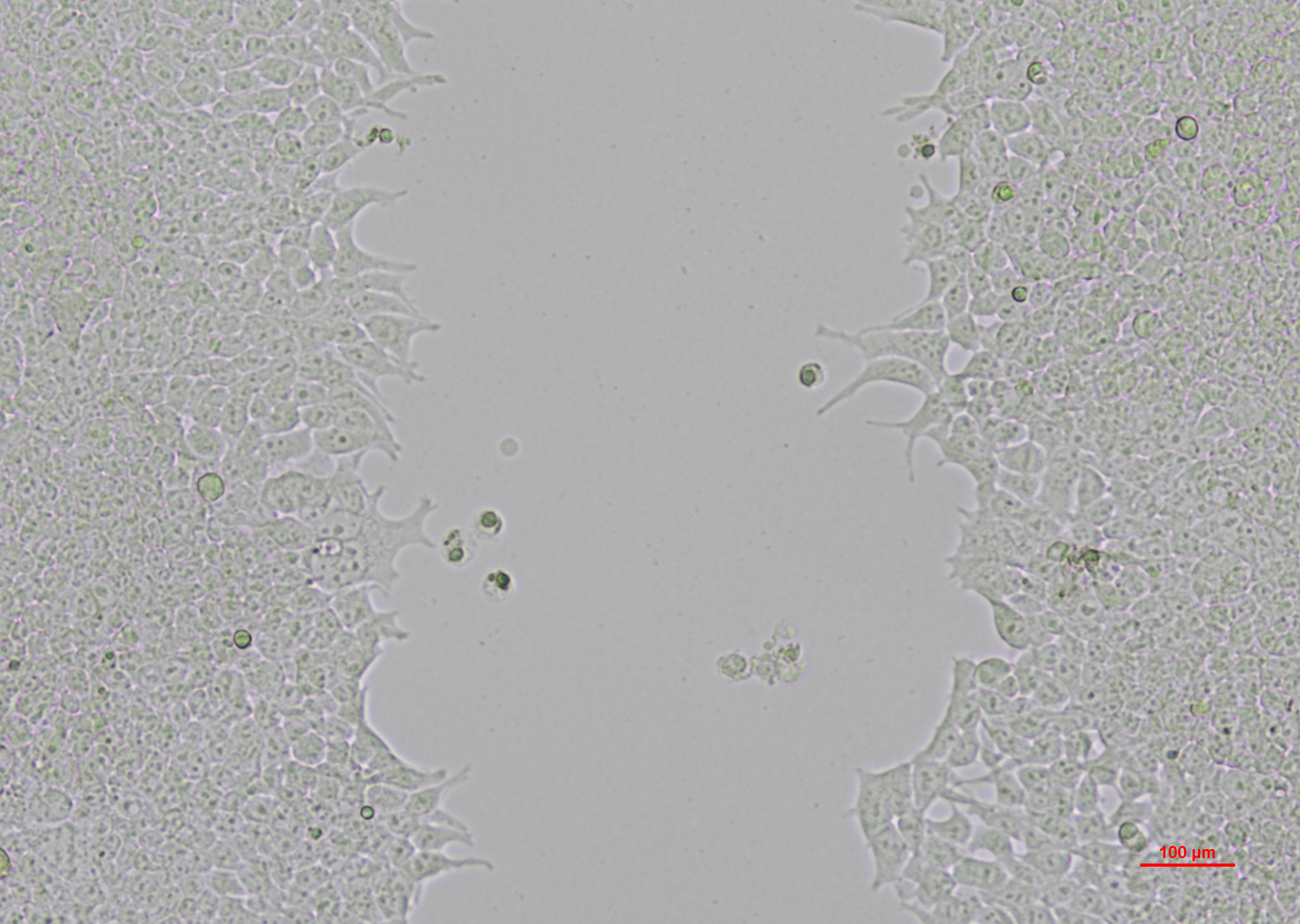

Supplement: Supplementary file 9 [file Image11.TIF]

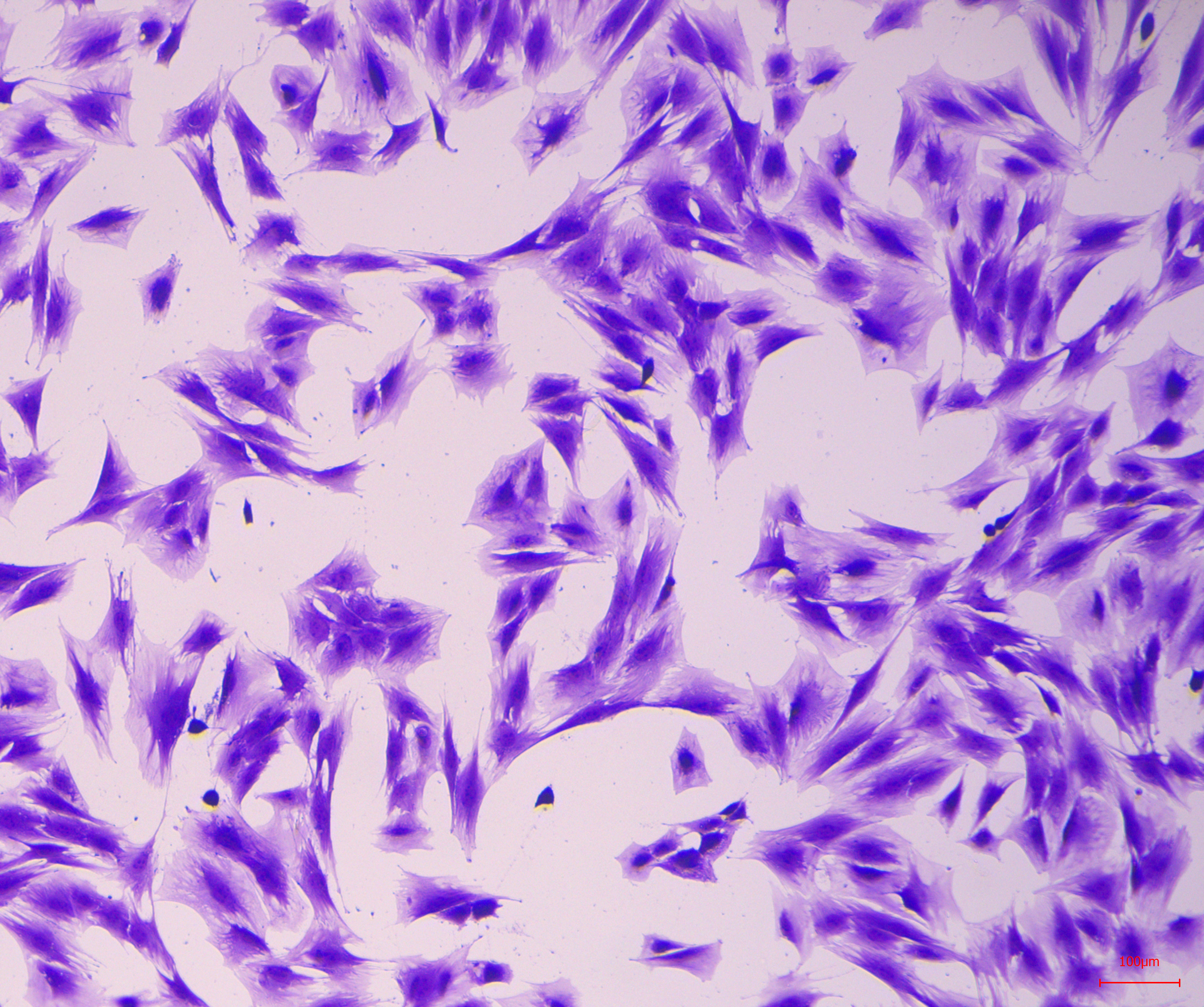

Supplement: Supplementary file 10 [file Image1.TIF]

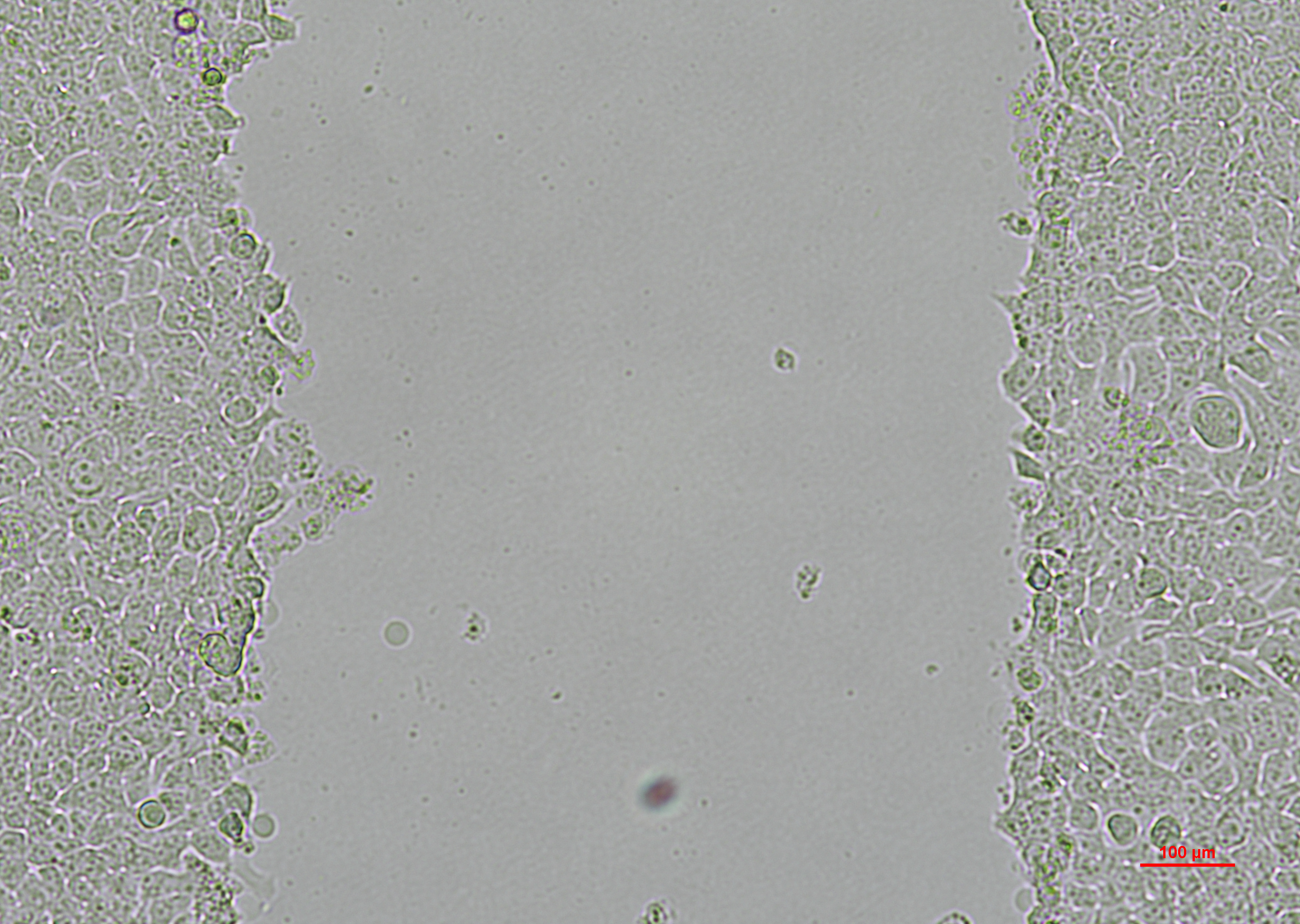

Supplement: Supplementary file 11 [file Image10.TIF]

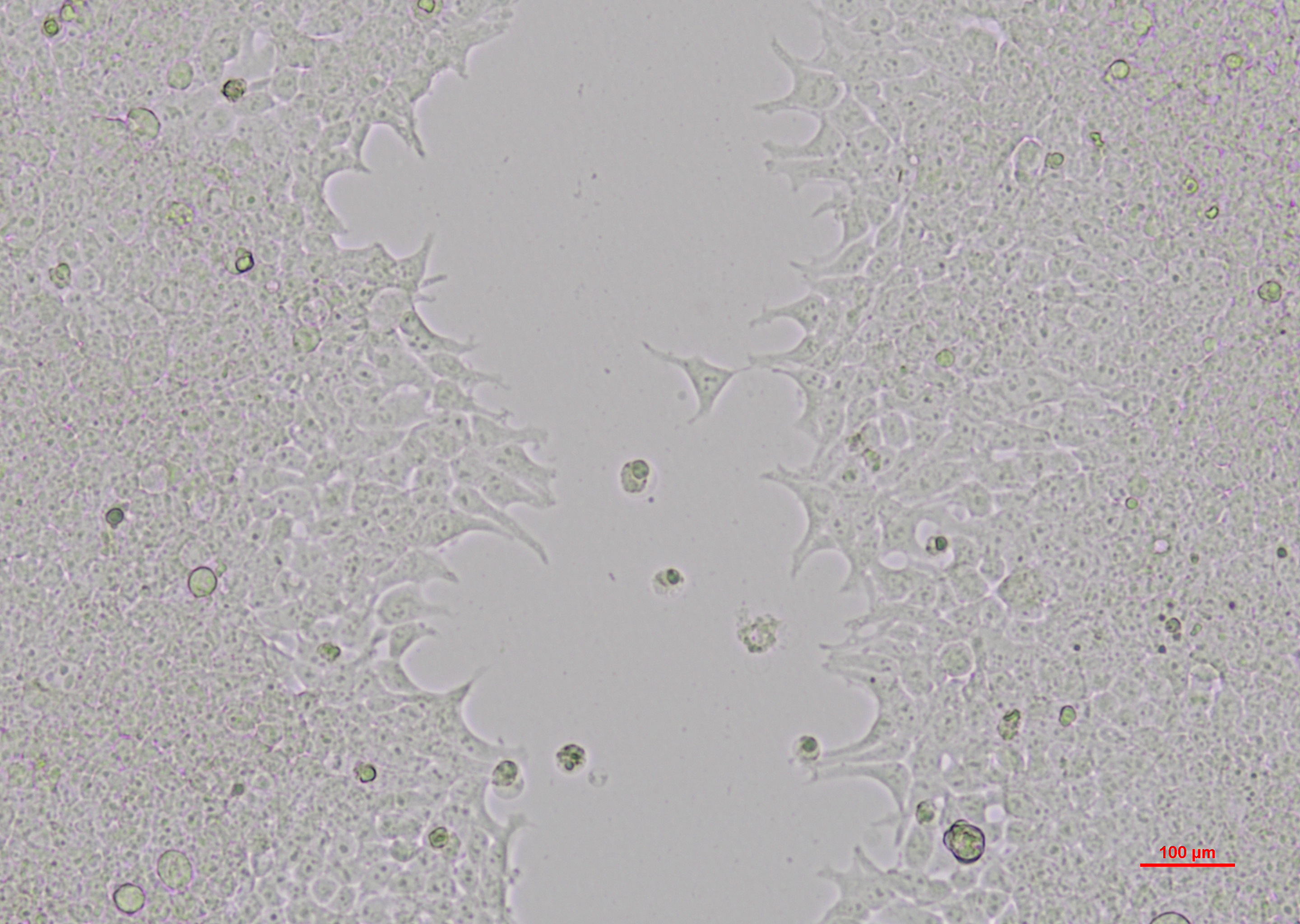

Supplement: Supplementary file 12 [file Image7.TIF]

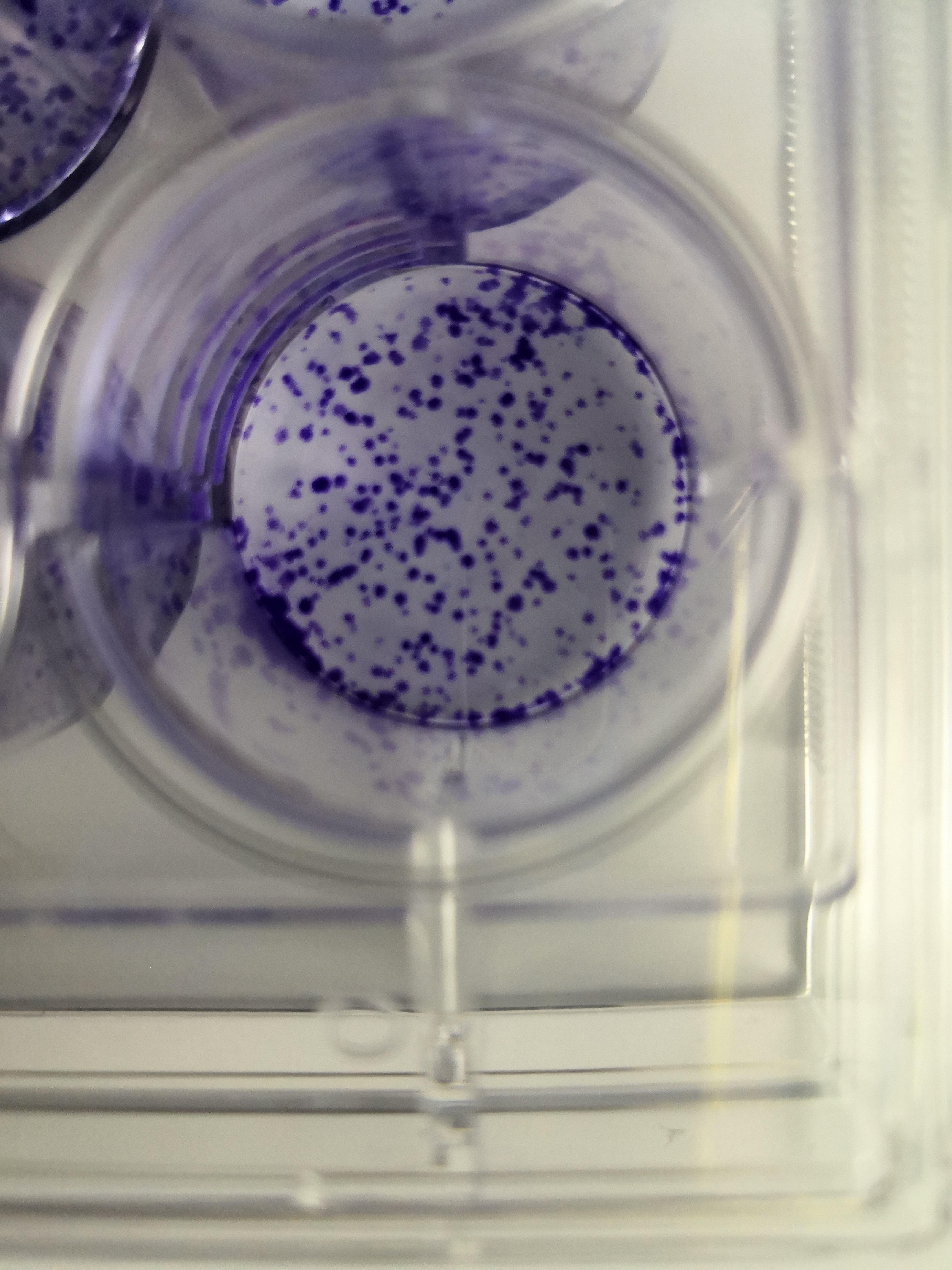

Supplement: Supplementary file 13 [file DataSheet2.ZIP › Cell cloning/DR group.jpg]

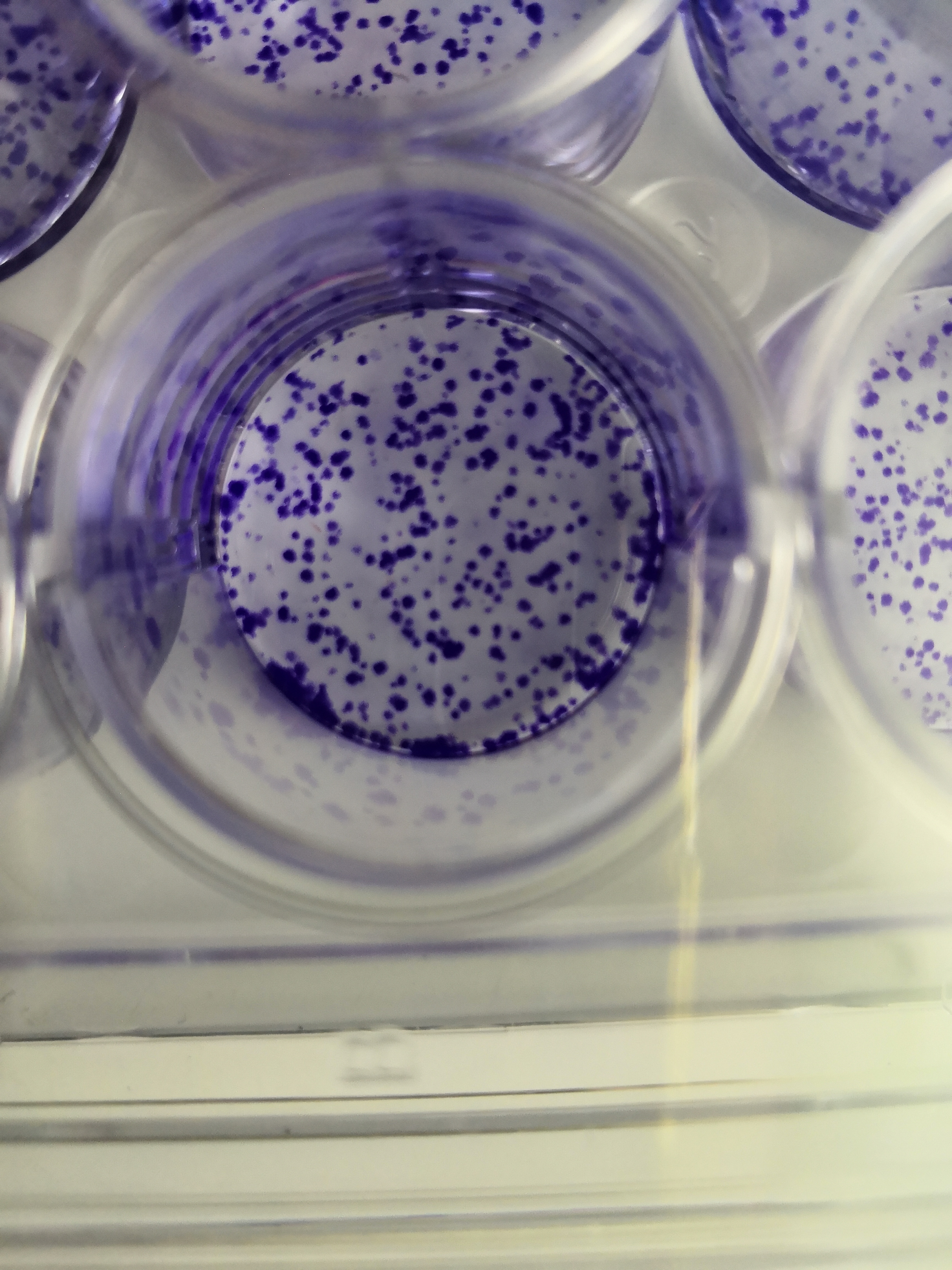

Supplement: Supplementary file 13 [file DataSheet2.ZIP › Cell cloning/DR+mimic-NC group.jpg]

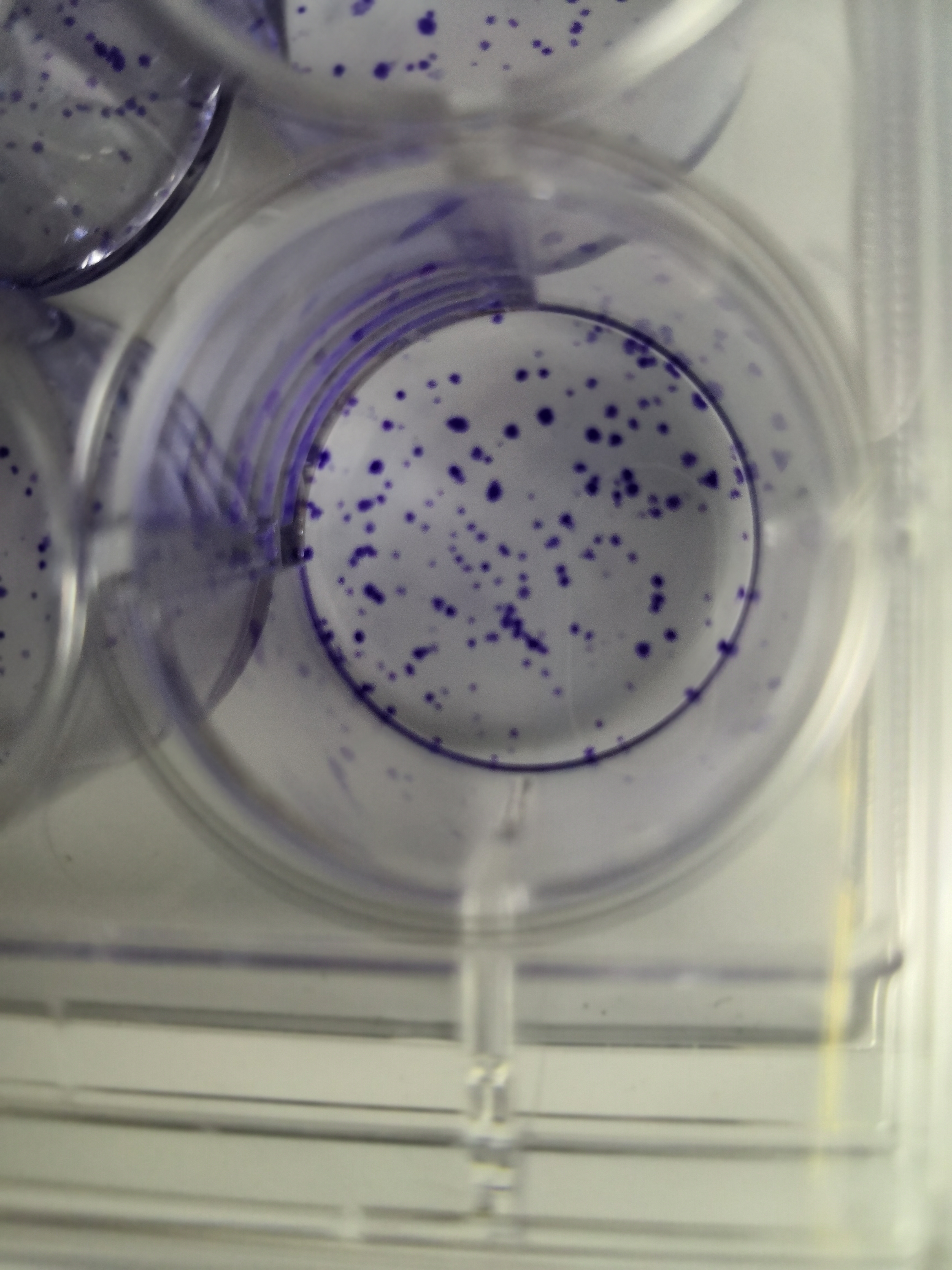

Supplement: Supplementary file 13 [file DataSheet2.ZIP › Cell cloning/DR+miR-103a-3p-mimic group.jpg]

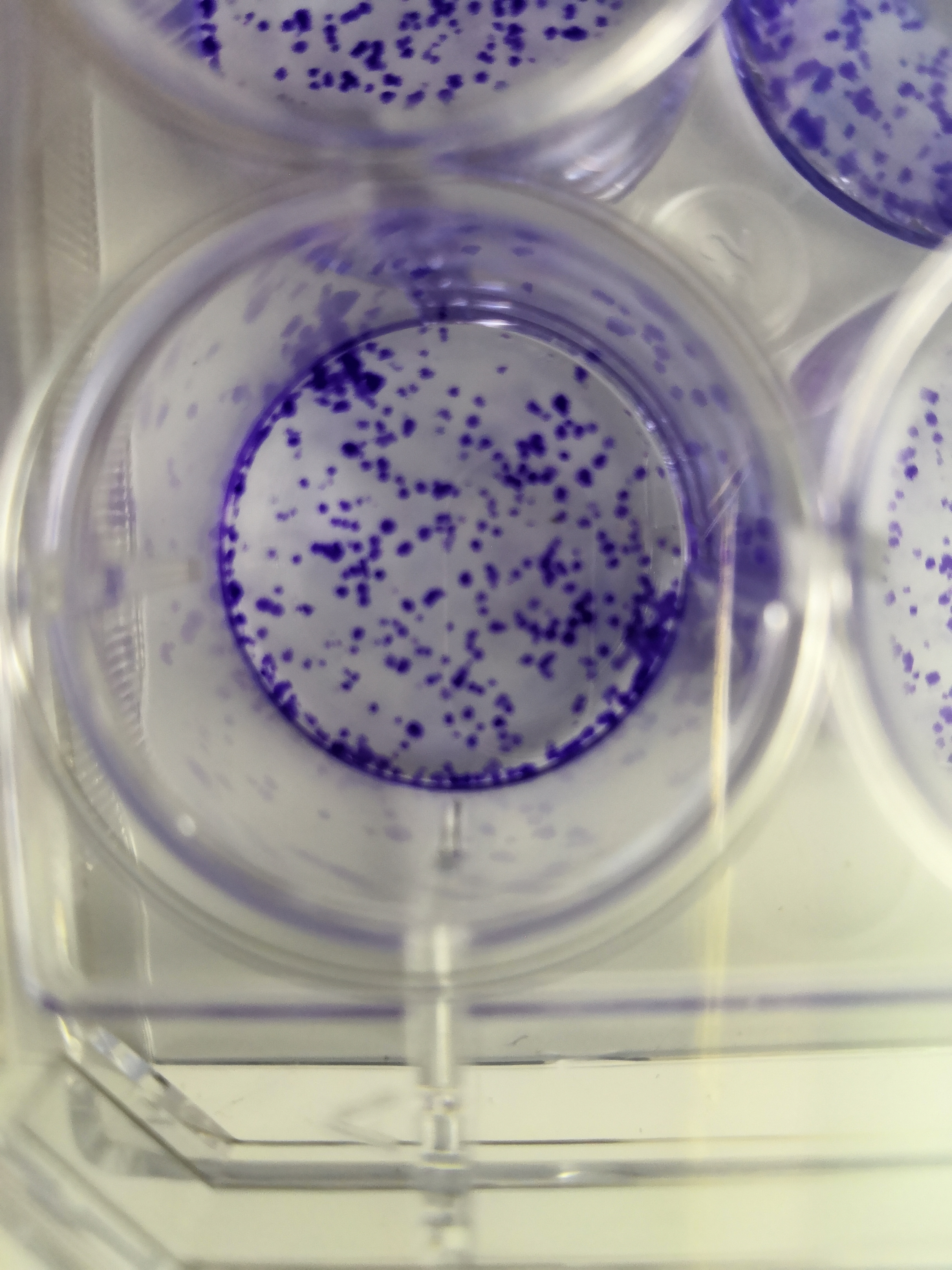

Supplement: Supplementary file 13 [file DataSheet2.ZIP › Cell cloning/DR+si-NC group.jpg]

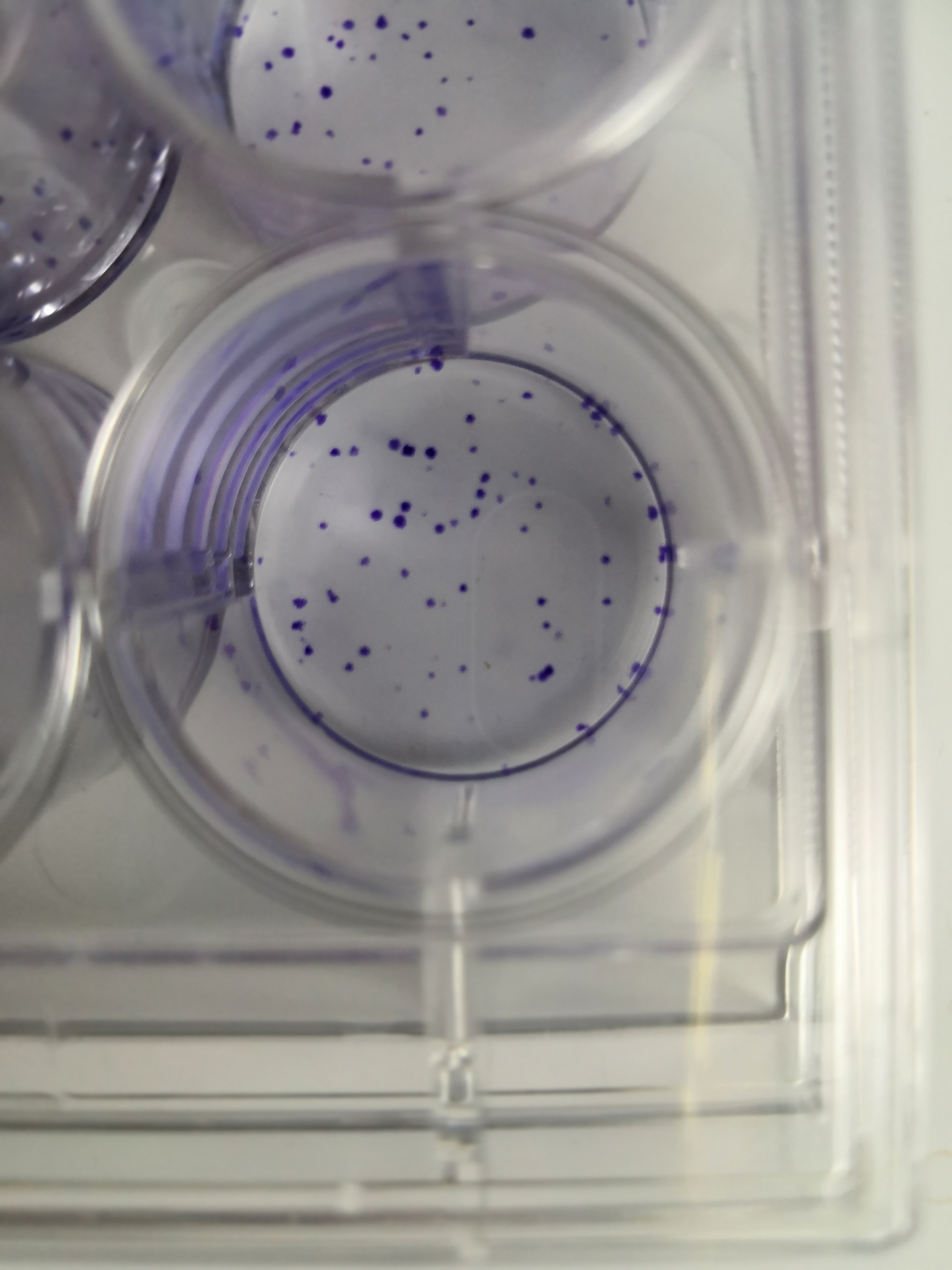

Supplement: Supplementary file 13 [file DataSheet2.ZIP › Cell cloning/DR+si-TRIM66 group.jpg]

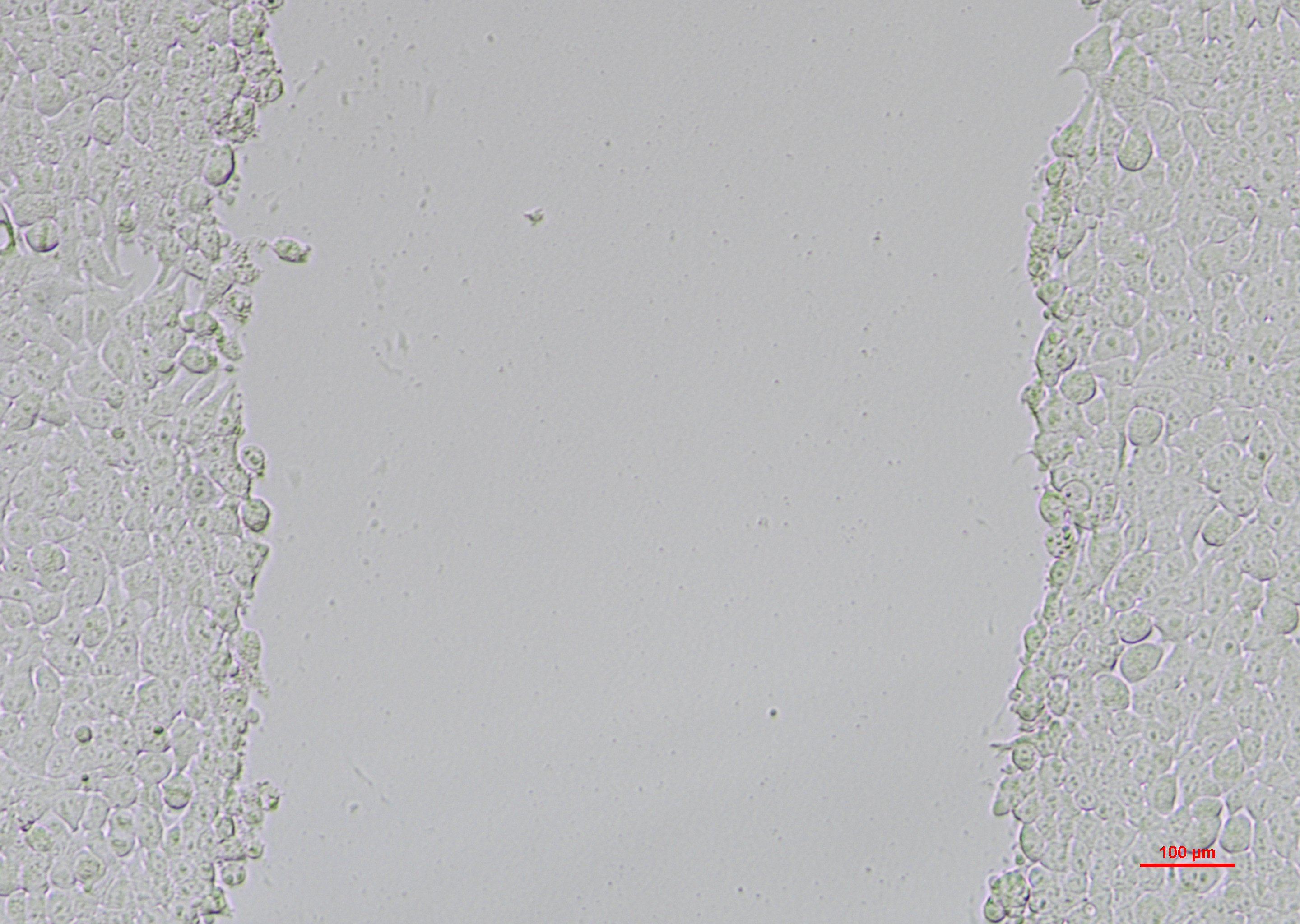

Supplement: Supplementary file 15 [file Image8.TIF]

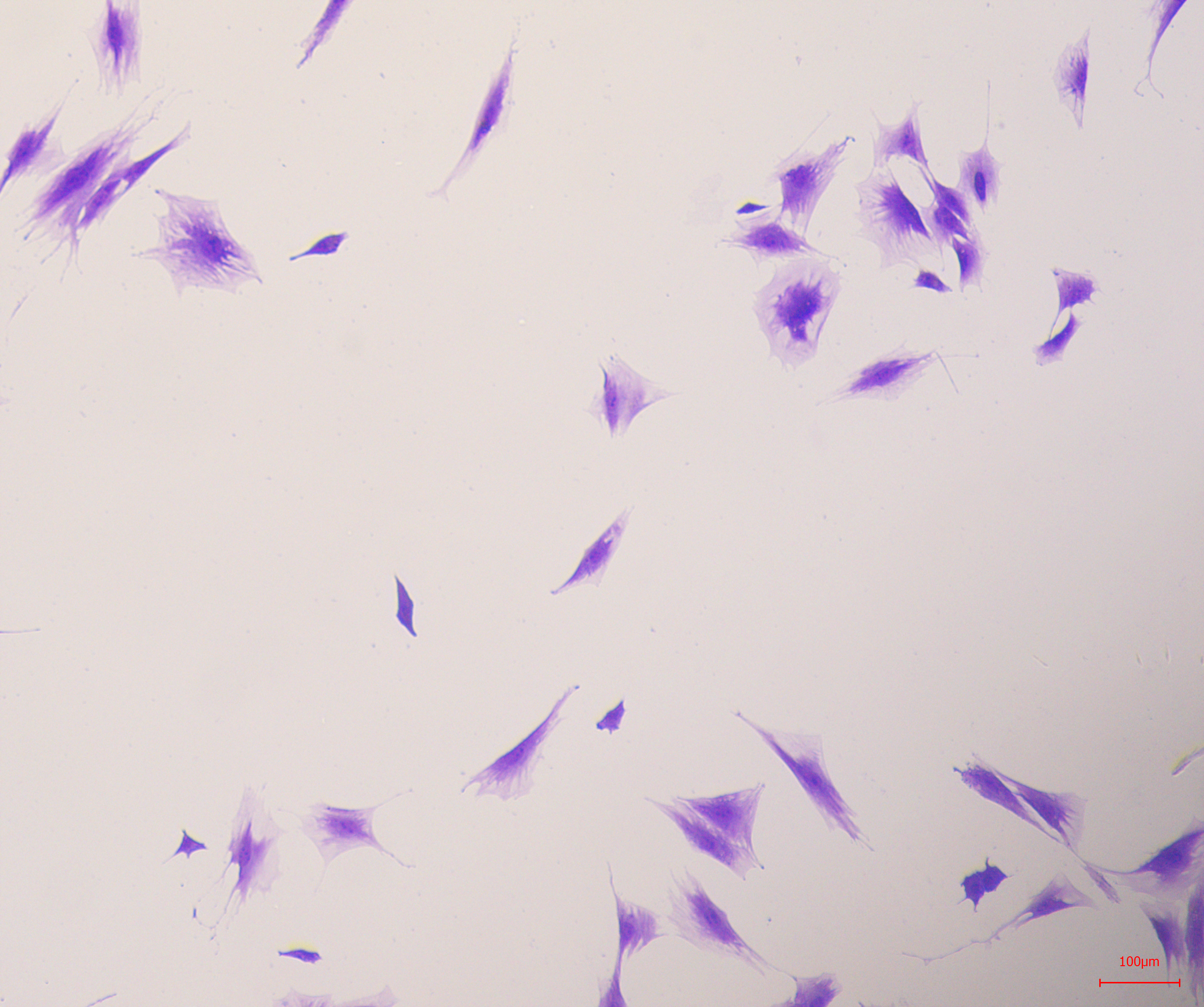

Supplement: Supplementary file 16 [file Image5.TIF]

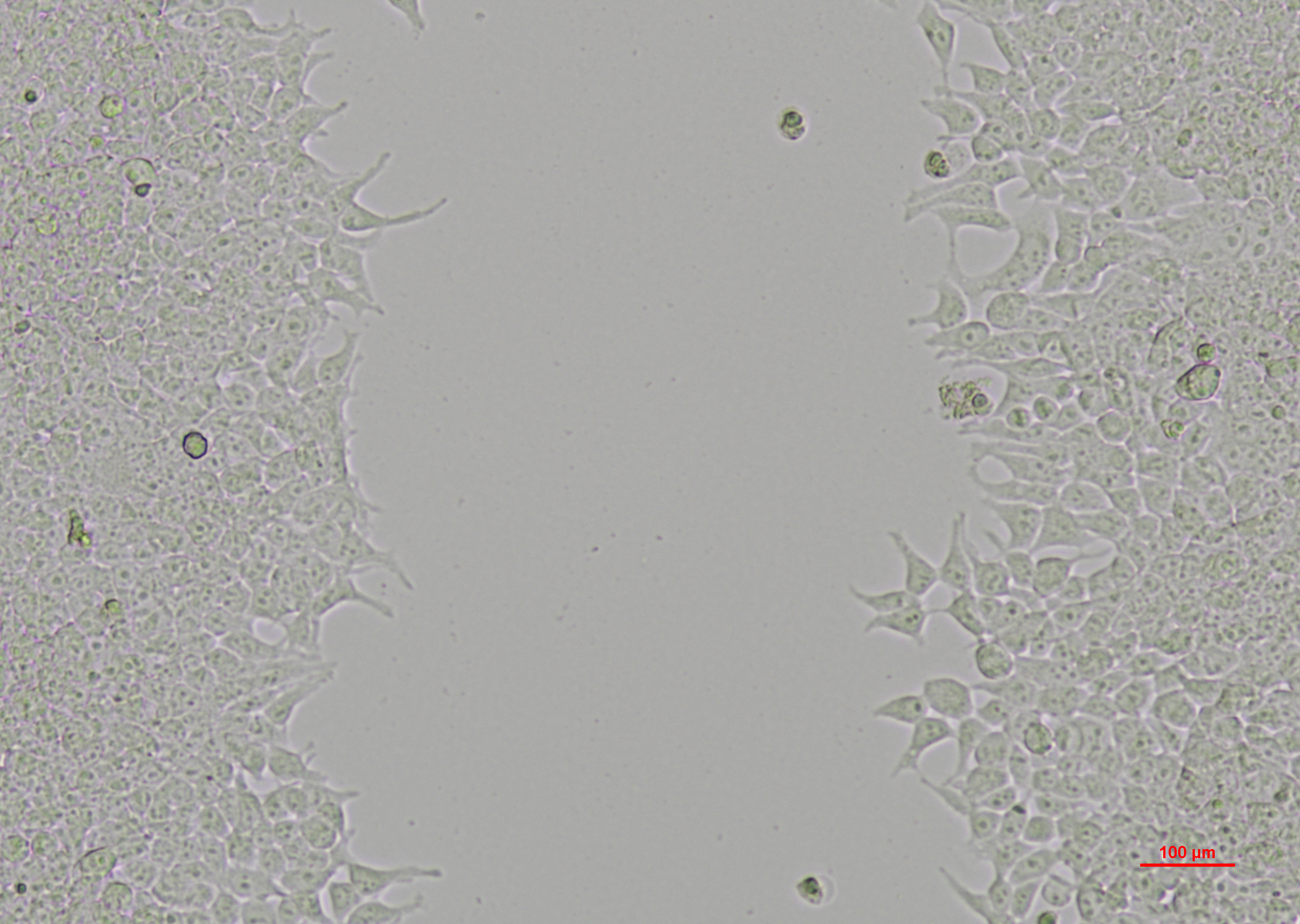

Supplement: Supplementary file 17 [file Image15.TIF]

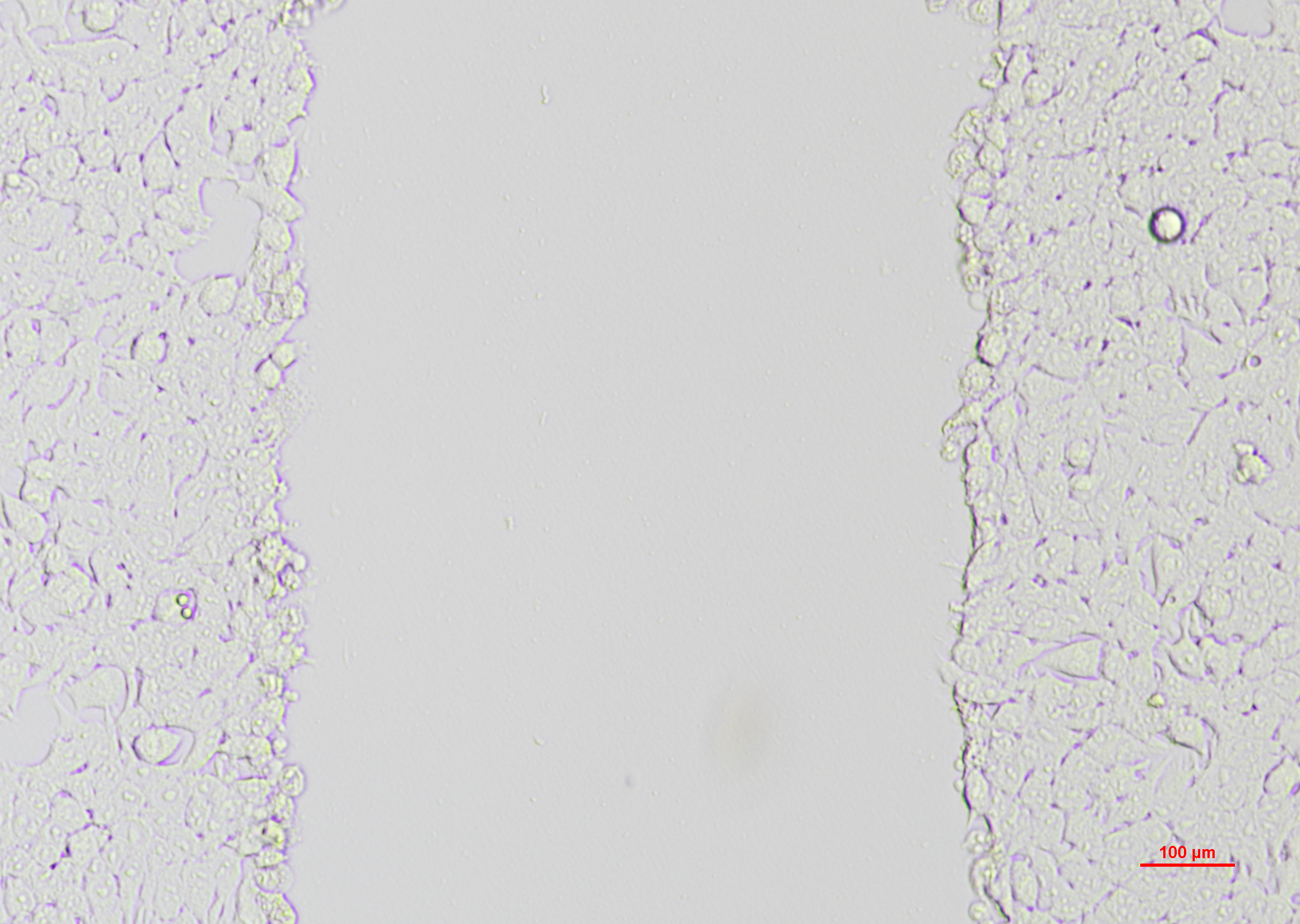

Supplement: Supplementary file 18 [file Image12.TIF]
